# SARS-CoV-2 infection and viral fusogens cause neuronal and glial fusion that compromises neuronal activity

**DOI:** 10.1126/sciadv.adg2248

**Published:** 2023-06-07

**Authors:** Ramón Martínez-Mármol, Rosina Giordano-Santini, Eva Kaulich, Ann-Na Cho, Magdalena Przybyla, Md Asrafuzzaman Riyadh, Emilija Robinson, Keng Yih Chew, Rumelo Amor, Frédéric A. Meunier, Giuseppe Balistreri, Kirsty R. Short, Yazi D. Ke, Lars M. Ittner, Massimo A. Hilliard

**Affiliations:** ^1^ Clem Jones Centre for Ageing Dementia Research, Queensland Brain Institute, The University of Queensland, Brisbane, QLD 4072, Australia.; ^2^ Dementia Research Centre, Macquarie Medical School, Faculty of Medicine, Health and Human Sciences, Macquarie University, Sydney, NSW 2109, Australia.; ^3^ School of Chemistry and Molecular Biosciences, Faculty of Science, The University of Queensland, Brisbane, QLD 4072, Australia.; ^4^ Queensland Brain Institute, The University of Queensland, Brisbane, QLD 4072, Australia.; ^5^ School of Biomedical Sciences, The University of Queensland, Brisbane, QLD 4072, Australia.; ^6^ Department of Virology, Faculty of Medicine, University of Helsinki, Helsinki FIN-00014, Finland.

## Abstract

Numerous viruses use specialized surface molecules called fusogens to enter host cells. Many of these viruses, including the severe acute respiratory syndrome coronavirus 2 (SARS-CoV-2), can infect the brain and are associated with severe neurological symptoms through poorly understood mechanisms. We show that SARS-CoV-2 infection induces fusion between neurons and between neurons and glia in mouse and human brain organoids. We reveal that this is caused by the viral fusogen, as it is fully mimicked by the expression of the SARS-CoV-2 spike (S) protein or the unrelated fusogen p15 from the baboon orthoreovirus. We demonstrate that neuronal fusion is a progressive event, leads to the formation of multicellular syncytia, and causes the spread of large molecules and organelles. Last, using Ca^2+^ imaging, we show that fusion severely compromises neuronal activity. These results provide mechanistic insights into how SARS-CoV-2 and other viruses affect the nervous system, alter its function, and cause neuropathology.

## Teaser

Viral infection and the expression of viral fusogens in the nervous system drive neuronal fusion compromising neuronal activity.

## INTRODUCTION

Infectious diseases that involve the nervous system are caused by a wide spectrum of agents, including bacteria, fungi, parasites, and viruses (*1*). Viruses from diverse families, such as rabies virus, herpes simplex virus, Epstein-Barr virus, Zika virus, reovirus, and severe acute respiratory syndrome coronavirus 2 (SARS-CoV-2) can infect neurons (*2*–*8*). Viral brain infections are characterized by multiple neurological symptoms, including headache, fever, confusion, epileptic seizures, and loss of taste or smell. In more severe cases, viral brain infections can lead to encephalitis and meningitis, as well as potentially irreversible neuronal deficits such as paralysis and death. Clinical symptoms can originate from the loss of infected neurons (*9*); however, some viruses do not kill their host cells, and the chronic neurological sequelae of these infections cannot be explained by neuronal death (*10*). Other neuropathological mechanisms must therefore underlie the progression of these viral infections, leading to brain dysfunction. In non-neuronal tissues, enveloped viruses and reoviruses use specialized molecules called fusogens to fuse with host membranes and enter cells (*11*). These viruses then hijack the cellular machinery to produce viral components, with newly synthesized viral fusogens redecorating the cell membrane and conferring the ability to fuse with neighboring cells. This results in the formation of multinucleated syncytia, which allow viral propagation “from within,” without the need for virion release into the extracellular space (*12*–*14*). As defined more than 100 years ago by Santiago Ramón y Cajal, the nervous system is composed of discrete neurons that act as individual units and do not base their development or communication on cellular fusion. Preserving neuronal individuality is critical for the correct function of the nervous system, and it is still poorly understood whether viral infection and the resulting presence of viral fusogens can cause neuronal fusion and the formation of syncytia, thereby permanently altering the neuronal circuitry and function.

## RESULTS

### SARS-CoV-2 infection causes neuron-neuron, neuron-glia and glia-glia fusion in murine hippocampal cultures and in human-derived brain organoids

**Fig. 1.**
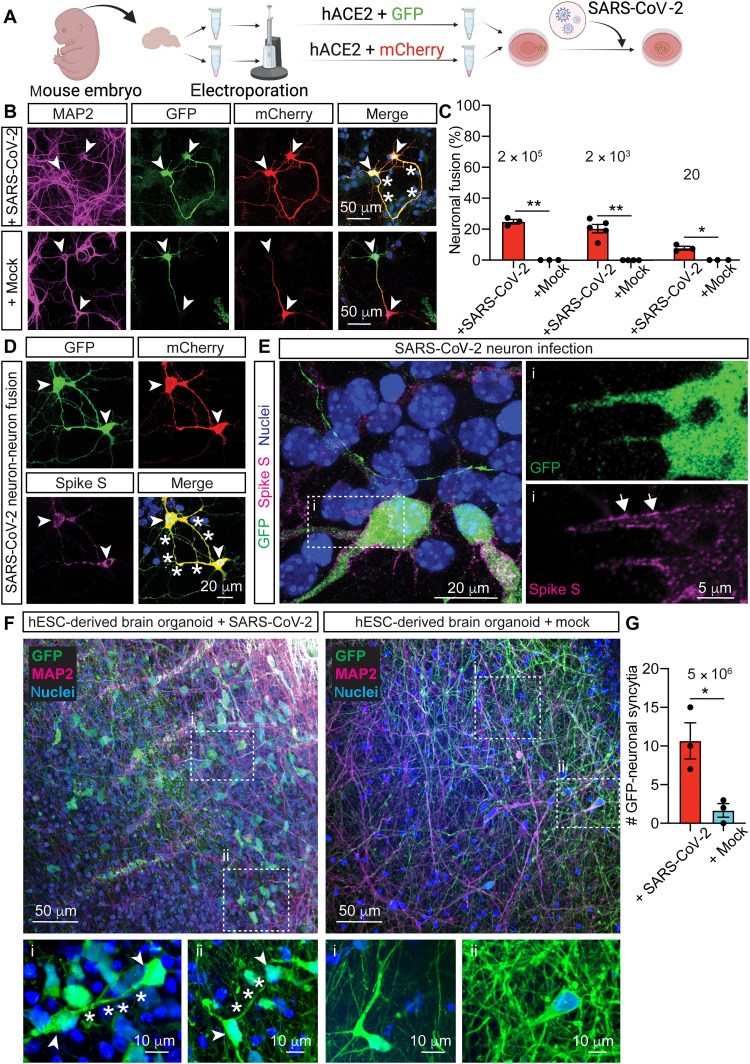
SARS-CoV-2 infection induces neuronal fusion. (**A**) Scheme of the fluorescence fusion assay. Two independent populations of hippocampal neurons were electroporated with either hACE2 and GFP or hACE2 and mCherry. After electroporation, the neurons were cultured together and 5 days later were infected with SARS-CoV-2 or mock control. (**B**) Representative images of SARS-CoV-2–infected fused neurons (top row) identifiable by GFP (green) and mCherry (red) appearing in both neurons (yellow in merge), and nonfused mock control (bottom row). (**C**) Quantification of neuronal fusion expressed as the percentage of fused neurons out of the total fluorescent neurons in close proximity (< 200 μm). (**D**) Representative images of fused neurons positive for spike S staining. (**E**) Representative images showing the distribution of spike S in infected neurons. The boxed area (i) is at higher magnification in the right panels, with GFP (top) and spike S (bottom) distributed along the surface of the infected neuron (arrows). (**F**) Representative images of hESC-derived brain organoids infected with SARS-CoV-2 (left) or with mock control (right). Boxed areas (i and ii) are magnified in the bottom panels, with examples of GFP-neurons (arrowheads) fused at their neurites (asterisks). (**G**) Quantification of neuronal syncytia per organoid. In (B) and (F), immunocytochemistry shows nuclei (blue), neuronal MAP2 (magenta), GFP (green), and mCherry (red). In (D) and (E), immunocytochemistry shows nuclei (blue), GFP (green), mCherry (red), and SARS-CoV-2 spike S (magenta). In all images, arrowheads indicate somas, asterisks indicate fused neurites, and arrows indicate spike S protein on the membrane. Data in (C) and (G) represent means ± SEM. In (C), *n* = 3 independent infections, with three dishes per infection, and 19 to 60 neurons per dish. In (G), *n* = 3 brain organoids were infected. Unpaired two-tailed Welch’s *t* tests were used in (C) and (G). *P ≤ 0.05 and **P < 0.01.

SARS-CoV-2 causes primarily a respiratory disease, but increasing evidence has revealed the presence of viral RNA and proteins also in the brain and a multitude of neuropsychiatric syndromes (*8*, *15*, *16*), which appear in the early stages of the disease and persist for months after infection, in what has recently been termed long COVID (*17*). Viral fusogens are involved in the recognition, binding, and entrance of viruses into their host cells. During infection, these proteins are expressed de novo to form new viral particles, trafficking from secretory organelles to the host plasma membrane and causing cell-cell fusion (*18*, *19*). To determine whether viral neuroinfection induces the fusion of neurons, we used SARS-CoV-2 and a fluorescence fusion assay, whereby different intracellular fluorophores transfer between fused cells. SARS-CoV-2 uses the human angiotensin-converting enzyme 2 (hACE2) as its main receptor on the surface of the host cell (*20*), and neuropilin 1 (NRP1) as a cofactor to enhance infectivity (*21*). Among other tissues, hACE2 is expressed in neuronal and glial cells in the human central nervous system (*22*). Mouse neurons express mACE2 (*23*), which shares 81.86% interspecies homology with the human protein but lacks key residues for spike S binding (*20*), resulting in modest to weak affinity for SARS-CoV-2 (*24*). We, therefore, expressed hACE2 in murine-derived brain cells. Immediately after embryonic hippocampal dissection, a population of these brain cells was electroporated with a plasmid encoding hACE2 and another encoding green fluorescent protein (GFP); a second population of hippocampal cells was electroporated with a plasmid encoding hACE2 and another encoding mCherry. The two neuronal populations were then plated together and maintained for 5 days in vitro (5 DIV; Fig. 1A). The original ancestral SARS-CoV-2 was amplified in Vero cells expressing human transmembrane protease serine 2 (TMPRSS2) and titrated by plaque assay (*25*). The neuronal cultures were infected with 2 × 10^5^, 2 × 10^3^, or 20 plaque-forming units (PFUs) or were mock-infected with control culture medium. Seventy-two hours post-infection (hpi), the cultures were fixed and examined by confocal fluorescence microscopy, revealing the presence of fused neurons [positive for the neuronal marker microtubule-associated protein 2 (MAP2)], as characterized by the presence of both the GFP and mCherry fluorescent proteins for all the SARS-CoV-2 titers used (Fig. 1, B and C). Antibody staining revealed that fused neurons were positive for the fusogenic spike S protein (Fig. 1D and fig. S1, A and B) (*26*), which was distributed across the surface of infected neurons (Fig. 1E). In contrast, neuronal fusion was not observed in the mock control, and the spike S protein was not detected (Fig. 1B and fig. S1, A and B). Upon SARS-CoV-2 infection, glial cells expressing hACE2 were also positive for the spike S protein (fig. S1, C and D), and we observed additional fusion phenotypes, including neuron-glia (fig. S2, A to C) and glia-glia fusion (fig. S2, D to F). In agreement with previous findings (*2*), we found that high doses of neuronal SARS-CoV-2 infection resulted in cell damage, a phenotype that was not apparent at the lowest doses used within 72 hpi (fig. S1, E and F).

We next investigated whether viral infection induced the fusion of human neurons by infecting human embryonic stem cell (hESC)–derived three-dimensional (3D) brain organoids with SARS-CoV-2. Such organoids have become one of the preclinical models of choice to study SARS-CoV-2 pathogenesis (*27*) and have been used to demonstrate that the virus can infect human neurons with endogenous expression of ACE2 (*8*, *27*–*30*). To visualize whether organoid infection induced neuronal fusion, 43- to 50-day-old brain organoids were generated using a modification of previously reported protocols (*31*) and were transduced with adeno-associated virus (AAV) expressing GFP. This resulted in the stable expression of GFP in sparse neurons. The transduced organoids were then cultured for an additional 10 days before being infected with SARS-CoV-2 or with mock control and were fixed 72 hpi. Similar to what was observed after infection of 2D neuronal cultures, we found that SARS-CoV-2–infected brain organoids exhibited neuronal syncytia formed by GFP-interconnected neurons (Fig. 1, F and G).

### Viral fusogens cause neuron-neuron, neuron-glia, and glia-glia fusion

**Fig. 2.**
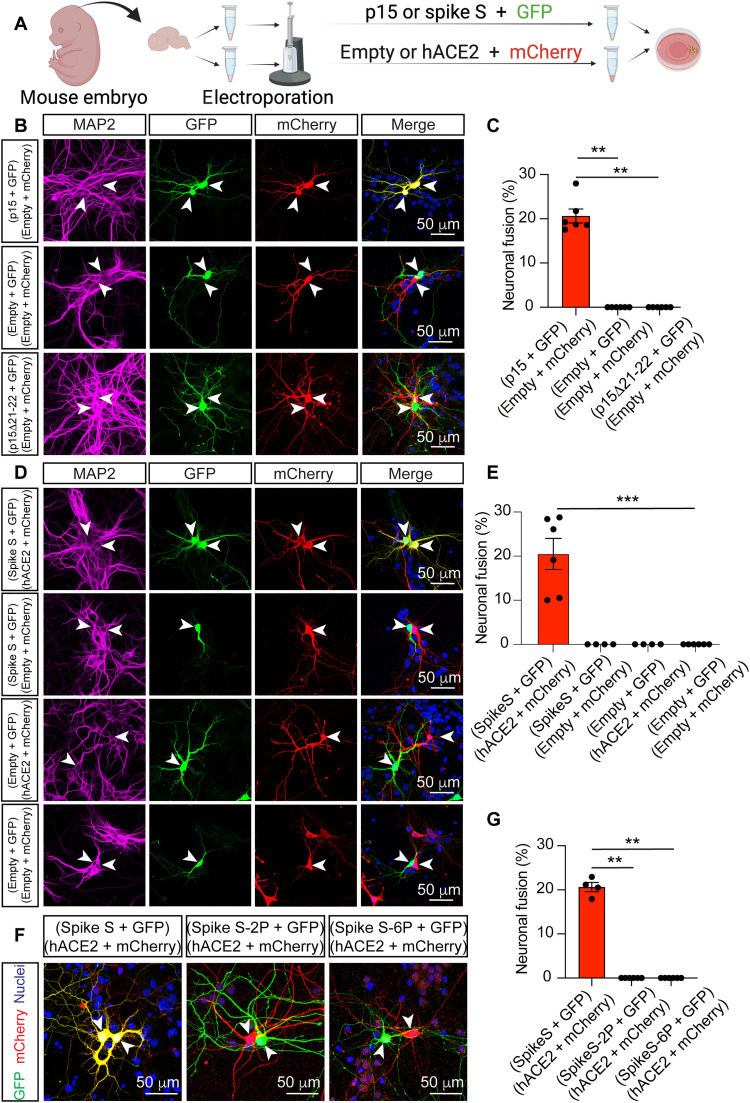
Expression of the viral fusogens p15 and spike S is sufficient to induce neuronal fusion. (**A**) Scheme of the fluorescence fusion assay. Two independent populations of hippocampal neurons were electroporated with either fusogen (p15 or spike S) and GFP, or empty vector or hACE2 and mCherry. After electroporation, the neurons were cultured together for 7 days. (**B**) Representative images of fused neurons (top row) identifiable by GFP (green) and mCherry (red) fluorescence appearing simultaneously in adjacent neurons (yellow in merge) and nonfused control neurons (middle and lower rows). Fusion was observed upon expression of p15 but not with the nonfusogenic mutant p15Δ21-22 or by the empty vector. (**C**) Quantification of neuronal fusion as the percentage of transfected neurons that fuse out of the total number of fluorescent neurons in close proximity (≤ 200 μm). (**D**) Representative images of fused neurons induced by the expression of spike S and hACE2 (top row) and nonfused control neurons (remaining three rows). (**E**) Quantification of neuronal fusion as the percentage of transfected neurons that fuse out of the total number of fluorescent neurons in close proximity (≤ 200 μm). (**F**) Representative images showing the lack of fusion associated with the inactive spike S mutants S-2P and S-6P, compared to the WT. (**G**) Quantification of neuronal fusion as the percentage of transfected neurons that fuse (yellow) out of the total number of fluorescent neurons in close proximity (≤ 200 μm). Images in (B), (D), and (F) show immunocytochemistry for nuclei (blue), neuronal MAP2 (magenta), GFP (green), and mCherry (red). In all images, arrowheads indicate somas. Data in (C), (E), and (G) are displayed as means ± SEM; *n* > 150 neurons analyzed in four to six independent dishes from more than two cultures, one-way analysis of variance (ANOVA) Kruskal-Wallis test followed by Dunn’s post hoc test comparing all groups to the empty vector control. **P < 0.01 and ***P < 0.001.

To study whether and how the mere presence of viral fusogens on the surface of host cells affect the nervous system, we used the spike S protein of SARS-CoV-2 and the p15 fusogen isolated from the baboon orthoreovirus (BRV), which infects the brain of these primates, causing meningoencephalomyelitis (*5*, *32*). p15 is a fusion-associated small transmembrane fusogen. Unlike the spike S protein, p15 is the only viral protein required by the BRV to form a syncytium (*33*), with no receptor protein on the host cell being needed to mediate fusion (*34*, *35*). We first expressed p15 in embryonic mouse primary hippocampal neurons and visualized the presence of fusion through the fluorescence fusion assay described above. Immediately after isolation, a population of neurons was cotransfected by electroporation with a plasmid containing p15 and another containing GFP; a second population of neurons was cotransfected with a plasmid containing mCherry and an empty control vector. The two neuronal populations were then plated together and maintained in culture for 7 DIV (Fig. 2A). Our results revealed that the expression of p15 was sufficient to induce neuronal fusion, as detected by the presence of neurons containing both the GFP and mCherry fluorescent proteins [Fig. 2, B (first row) and C], a phenotype that was never observed when the control vector was cotransfected in the absence of p15 [Fig. 2, B (second row) and C]. To determine whether the fluorophore diffusion was caused by the fusogenic properties of p15, we generated an inactive version of this fusogen, p15Δ21-22, in which two residues of the N terminus of the transmembrane domain were truncated (*36*). Expression of this inactive fusogen completely abolished neuronal fusion [Fig. 2, B (third row) and C].

Unlike p15, the spike S protein must bind to the hACE2 receptor to trigger fusion, requiring both spike S and hACE2 to be expressed to promote neuronal fusion mediated by the viral fusogen. Using a similar approach to that described for p15, we electroporated two neuronal populations, one with a plasmid expressing GFP plus a plasmid containing a codon-optimized version of the spike S protein (*37*) and the other with a plasmid expressing mCherry plus a plasmid containing the hACE2 receptor. The two populations were then plated together and cultured for 7 DIV. The expression of the fusogen spike S and its receptor hACE2 in adjacent cells resulted in the fusion of these neurons and the mixing of the fluorescent proteins [Fig. 2, D (first row) and E]. The presence of both the fusogen and its specific receptor was required to initiate cellular fusion, as the expression of either spike S or hACE2 alone did not generate any fusion events [Fig. 2, D (second and third rows) and E]. To determine whether the fusion of neurons was caused by the fusogenic properties of spike S, we used two fusion-inactive versions of this protein, spike S-2P and spike S-6P (HexaPro). We first generated the spike S-2P variant, which contains two consecutive proline substitutions (K986P and V987P) in the C-terminal S2 subunit (*38*). These two mutations retain spike S in a prefusion conformation, blocking its fusion capacity (*39*). Spike S-6P contains four additional proline substitutions (F817P, A892P, A899P, and A942P) that further stabilize the prefusion conformation and increase protein expression and the ability to withstand heat stress (*40*). Our results reveal that neither of these versions of inactive spike S induces neuronal fusion (Fig. 2, F and G). Spike S maturation is driven by proteases, such as TMPRSS2. Viral entry and cell-to-cell fusion are enhanced by TMPRSS2 and NRP1 (*21*). Thus, we monitored their expression and detected both TMPRSS2 (fig. S3, A to C) and NRP1 (fig. S3, D to F) in our neuronal cultures, suggesting that these proteins could be involved in the brain infectivity and neuronal fusion induced by SARS-CoV-2. With both p15 and spike S, we observed not only neuron-neuron fusion but also neuron-glia and glia-glia fusion when the fusogens were expressed in these cell types (fig. S4), a result that mimics our observation with SARS-CoV-2 infection.

Neuronal fusion implies a temporary or permanent diffusion of cytoplasmic material between cells (*41*). To confirm that this was the case, we used a variation of our fluorescence fusion assay. We cotransfected neurons with the photoconvertible fluorescent protein Kaede, which shifts from green to red fluorescence upon illumination with ultraviolet (UV) light (350 to 400 nm), together with either p15 or spike S and hACE2. After identification of interconnected adjacent green fluorescent neurons, we photoconverted the green Kaede fluorophore by applying brief pulses of UV light in a small region of one neuron (donor). The newly generated red photoconverted Kaede molecules rapidly diffused to the adjacent neuron (acceptor; fig. S5, A and E and movie S1). This diffusion was measured as a decrease in the red fluorescence within the donor neurons (fig. S5, B and F), with a concomitant increase in the acceptor neurons (fig. S5, C, D, G, and H), thereby conclusively demonstrating the existence of an active cytoplasmic bridge between p15-fused neurons and between spike S–hACE2–fused neurons. In the absence of fusion, red Kaede remained confined within the photoconverted neuron (fig. S5, A, E, and I to K). The fused neurons retained their morphology, extended processes, and remained viable.

### Viral fusogens induce neuronal fusion in vivo and in hESC-derived neurons

To determine whether the presence of viral fusogens can induce neuronal fusion in vivo, we generated transgenic *Caenorhabditis elegans* strains in which p15 and GFP were expressed simultaneously under the control of the *mec-4* promoter (*Pmec-4::p15* and *Pmec-4::GFP*), which is active in the six mechanosensory neurons (ALM left and right, AVM, PVM, and PLM left and right; fig. S6A). Similar to what we observed in mammalian neurons in culture, no fusion events with nearby neurons or tissues were detected in control animals expressing GFP under the *mec-4* promoter, and GFP was exclusively confined to the mechanosensory neurons [fig. S6, A (i) and B]. In contrast, we observed the appearance of additional GFP-positive cells in the head, mid-body, and tail of animals expressing the p15 fusogen [strains carrying p15 transgene *vdEx1266* or *vdEx1268*; fig. S6, A (ii to v) and B]. On the basis of its stereotypic location and morphology, we identified ALN as the most prevalent additional GFP-positive neuron [fig. S6, A (ii) and C]. The ALN neurons are a pair of sensory neurons located in the tail of the animal that extend their axons in close association with the axons of the ipsilateral ALM mechanosensory neurons (*42*). Other frequently GFP-positive neurons were the pair of LUA interneurons [fig. S6, A (iii) and C], which are located in the tail of the animal and extend anterior neurites in close proximity with those of the PLM neurons and the PVD mechanosensory neurons [fig. S6, A (v) and C], which are positioned in the mid-body of the animal with two extensively branched dendrites and a long axon (*42*). Despite over 90% of the GFP-positive cells being neurons, we also identified fluorescence in the hypodermal cells [fig. S6, A (iv) and C], which have a glial-like function forming a tissue in which the PLM and ALM axons are embedded (*43*, *44*). Similar to mammalian neurons, the expression of the inactive fusogen p15Δ21-22 within the mechanosensory neurons (*Pmec-4::p15Δ21-22*) did not result in fusion with neurons or hypodermal cells (fig. S6B). A temporal analysis of neuronal fusion observed during different stages of *C. elegans* life cycle revealed that the percentage of animals showing additional GFP-fused cells increased during the larval stages (L1 to L3), remaining constant in adulthood (fig. S6C). During our analysis of p15 expressed in mechanosensory neurons, we observed additional phenotypes that included defects in axonal guidance and axonal maintenance (visible as axonal breakages), as well as loss of mechanosensory neurons (fig. S6, D and E).

Next, to determine whether fusion could occur in vivo in the mammalian brain, we expressed p15 in the brains of adult mice. We designed AAV vectors expressing either GFP alone (+empty) or GFP and p15 (+p15) under the control of the neuronal human *Synapsin 1* (*hSyn*) promoter. AAVs were bilaterally injected into the hippocampus and the cortex of 11-week-old wild-type (WT) mice. Seven and 14 days after the AAV injections, the brains were removed for immunohistochemical analysis of neurons expressing p15 (fig. S7). Similar to what we observed in neuronal cultures and in *C. elegans* mechanosensory neurons, p15 expression resulted in neuronal fusion presenting as clusters of interconnected GFP-positive cells (fig. S7, A to E, and movie S2). Neuronal fusion was observed both in the hippocampus and in the cortex (fig. S7, F to I), representing up to 15% of transduced hippocampal neurons at 14 days after AAV infection (fig. S7H). Moreover, neuronal fusion resulted in a significant increase of total GFP-positive neurons (fig. S7, G and I) arising from the diffusion of GFP into adjacent cells, a value that increased with transduction time from 7 to 14 days (fig. S7J).

We next sought to determine whether fusion could also be observed in human-derived neurons by expressing either p15 or spike S in two different hESC-derived neuronal systems: cortical neurons differentiated in 2D cultures and 3D brain organoids. We first differentiated cortical neurons from hESCs and neuronal progenitor cells for 40 to 50 DIV and cotransfected them with GFP and either p15, spike S, or the inactive spike S-6P. Three days after the expression of the fusogens, the neuronal cultures were inspected, revealing the formation of neuronal clusters of interconnected GFP-positive cells that resembled the neuronal syncytium observed after p15 expression in murine neurons (fig. S8, A to C). The fact that cellular clusters were also observed when the spike S protein was expressed is a direct indication that neuronal fusion can occur using the endogenous hACE2 receptors expressed in these human cells. In the absence of fusogens or in the presence of the spike S-6P inactive mutant, no cell fusion was observed (fig. S8, A to C). We then differentiated brain organoids from hESCs until 43 to 50 DIV and expressed a similar combination of plasmids, consisting of the fluorescent protein mCherry and either p15, spike S, or the inactive spike S-6P. We maintained the organoids for three additional days before imaging them. Increments in the formation of mCherry-positive cell clusters were only detected in response to the expression of p15 or spike S (fig. S8, D and E), with both the empty vector and the spike S-6P mutant failing to induce cell fusion (fig. S8, D and E).

### Neurons fuse at their neurites and exchange large organelles

**Fig. 3.**
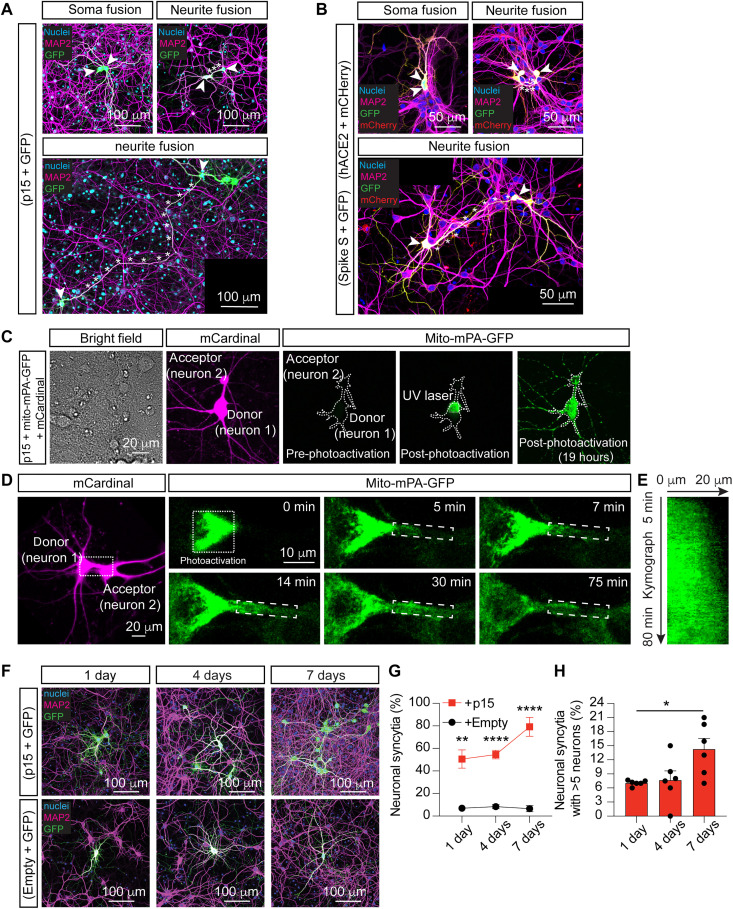
Fused neurons form long neuronal bridges and exchange large organelles. (**A**) Representative fused neurons (arrowheads) transfected with p15 and GFP. (**B**) Representative fused neurons (arrowheads) transfected with either spike S and GFP or hACE2 and mCherry before being cocultured. In both (A) and (B), fusion is observed at the somas (top left) or the neurites (top right and bottom), with neuronal bridges (asterisks) of variable lengths. Immunocytochemistry for nuclei (blue), MAP2 (magenta), GFP (green), and mCherry (red). (**C** and **D**) Representative fused hippocampal neurons cotransfected at 7 DIV with p15, mCardinal, and mito-mPA-GFP. (C) mito-mPA-GFP is caged and becomes visible only after UV photoactivation. In fused neurons, mitochondria diffuse from the donor to the acceptor neuron. (D) The boxed area is magnified in the time series panels, with photoactivated mito-mPA-GFP moving anterogradely along the neuronal bridge. (**E**) Kymograph of mito-mPA-GFP moving between fused neurons in (D). (**F**) Representative neurons fusing over time. Hippocampal neurons cotransfected at 7 to 10 DIV with p15 and GFP (top) or with empty vector and GFP in control (bottom) and cultured for 1, 4, or 7 days. Immunocytochemistry for nuclei (blue), MAP2 (magenta), and GFP (green). (**G**) Quantification of neuronal syncytia as the percentage of fused neurons out of the total number of GFP-positive neurons. (**H**) Quantification of the average number of neurons per syncytium (more than five neurons). In all images, arrowheads indicate somas and asterisks indicate fused neurites. Data in (G) and (H) are means ± SEM, *n* > 350 neurons analyzed in more than four independent dishes from four cultures. Two-way ANOVA in (G) followed by Geisser-Greenhouse correction and Šidák post hoc test comparing treatments (+empty vector versus +p15) within conditions (days in culture). One-way ANOVA Kruskal-Wallis test followed by Dunn’s post hoc tests was used in (H) to compare all groups to 1 day. *P < 0.05, **P < 0.01, and ****P < 0.0001.

In most tissues, syncytia are normally formed at the level of the cell bodies. However, the unique neuronal morphology with long processes prompted us to ask whether fusion can occur at the level of the neurites and, thus, at a distance from the neuronal cell bodies. We found that, upon SARS-CoV-2 infection, neuronal fusion occurred between the somas, as well as between the neurites far from the somas (Fig. 1D and fig. S9, A and B). Similarly, fusion driven by p15 and spike S resulted in fusion bridges of variable lengths that could extend over hundreds of micrometers (Fig. 3, A and B). This is remarkable, as it implies that neuronal fusion can easily be missed if searching for classical multinucleated syncytia. Next, we asked whether the observed fusion bridges allowed the exchange of cellular components larger than fluorescent proteins. To address this question, we performed a variation of the tethered fluorophore assay (*45*). As a consequence of cell-cell fusion, small cytoplasmic elements such as fluorophores and other soluble proteins can diffuse between cells, whereas larger organelles such as mitochondria remained tethered to the cellular microtubule cytoskeleton. We combined the expression of the soluble fluorescent protein mCardinal, with that of the photoactivatable mitochondrial marker mito-mPA-GFP, which allows visualization of mitochondrial movement. Our results reveal not only the presence of mCardinal in both fused neurons but also the movement of mitochondria between them (Fig. 3, C to E, and movie S3). To further confirm that the mitochondria movement was bidirectional, we tagged mitochondria with two different fluorophores, mito-mPA-GFP and mito-mPA-mCherry 1, revealing an exchange of these organelles between the fused neurons (fig. S10).

Last, we asked whether neuronal fusion was restricted to two adjacent neurons or if it was a propagating event that generated syncytia with a larger number of interconnected neurons. To address this possibility, we monitored neurons cotransfected with p15 and GFP for the appearance of syncytia over a 7-day period (Fig. 3F). Expressing GFP under the *CMV* promoter ensures that high levels of fluorescence are detected as early as 4 hours after transfection. In the absence of fusogen, the number of isolated GFP-expressing neurons remained constant; by contrast, in the presence of p15, the number of neuronal syncytia increased over time, appearing as clusters of interconnected GFP-positive neurons that progressively incorporated new cells (Fig. 3, F to H). Moreover, through live confocal imaging performed over a period of 50 hours, we observed the progressive appearance of GFP in surrounding neurons, revealing the occurrence of fusion events (movie S4). Similarly, after p15 expression in hESC-derived brain organoids, the mCherry-positive cells progressively organized into clusters and their numbers increased over time (fig. S11).

### Fused neurons exhibit compromised neuronal activity

**Fig. 4.**
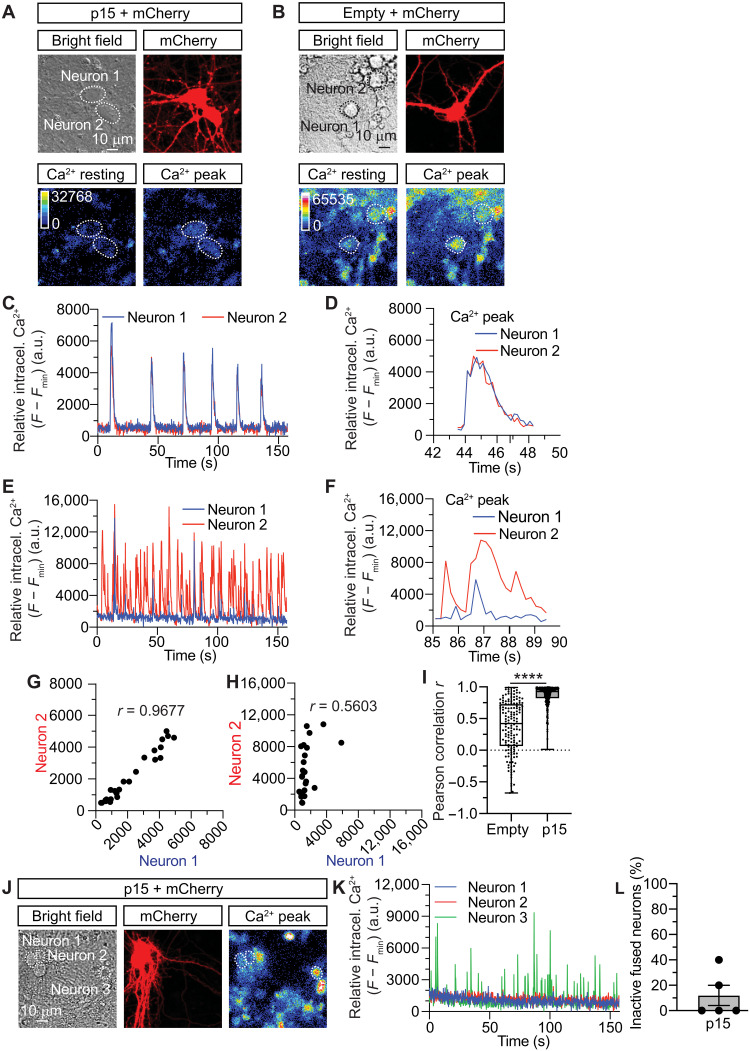
Neuronal fusion results in altered synaptic activity. (**A** and **B**), Representative hippocampal neurons (neuron 1 and neuron 2) cotransfected at 12 DIV with p15 and mCherry [(A), fused neurons] or with an empty control vector and mCherry [(B), nonfused neurons] and imaged 3 days later at 15 DIV after incubation with the Ca^2+^ indicator CAL-520. Bottom: Pseudocolored images of the intracellular Ca^2+^ levels in resting conditions and during an activity peak. (**C**) Plot of the relative intracellular Ca^2+^ intensity levels over time from fused neurons 1 and 2 of (A). (**D**) Detail of one of the intracellular Ca^2+^ peaks plotted in (C). (**E**) Plot of the relative intracellular Ca^2+^ intensity levels over time from nonfused neurons 1 and 2 of (B). (**F**) Detail of one of the intracellular Ca^2+^ peaks plotted in (E). (**G** and **H**) Scatterplot of Pearson’s correlation coefficient for the intracellular Ca^2+^ intensity levels of fused neurons (G) and nonfused neurons (H) of traces in (D) and (F), respectively. (**I**) Quantification of the average Pearson correlation (*s*) for the intracellular Ca^2+^ peaks from nonfused (empty) and fused (p15) neurons. (**J**) Images of two fused-but-inactive hippocampal neurons (neuron 1 and neuron 2) next to a nonfused active neuron (neuron 3). (**K**) Plot of the relative intracellular Ca^2+^ intensity levels of the fused neurons 1 and 2 and the nonfused neuron 3, from (J). Note the absence of intracellular Ca^2+^ peaks detected in neurons 1 and 2. (**L**) Percentage of inactive fused neurons. Data in (I) are means ± SEM. *n* = 147 Ca^2+^ peaks in nonfused (empty) and *n* = 318 Ca^2+^ peaks in fused (p15) neurons. Data in (L) are means ± SEM. All the data are obtained from >25 neurons imaged in five independent experiments. Unpaired two-tailed Welch’s *t*-test was used in (I). ****P < 0.0001. a.u., arbitrary units.

Our results suggest that fused neurons remain viable during long periods of time. However, whether fusion has an effect on neuronal activity remains unknown (*46*). To answer this question, we fused differentiated neurons (14 to 18 DIV) using p15 and visualized spontaneous neuronal activity with the fluorescent Ca^2+^-sensitive indicator Cal-520. Individual neurons expressing mCherry and the control empty vector exhibited spontaneous activity (fig. S12, A and B), an effect that was not observed in glial cells (fig. S12, C and D). The majority (~90%) of fused neurons resulted in synchronized neuronal activity (Fig. 4, A and C, and movie S5), with overlapping of Ca^2+^ peaks (Fig. 4, D, G, and I), whereas the remaining 10% displayed a complete loss of neuronal activity. A close evaluation of the latter subpopulation of cells revealed that it corresponded to those neurons that were fused tightly at the level of their somas (Fig. 4, J to L). In contrast, nonfused neurons presented a variable pattern of neuronal activity (Fig. 4, B and E, and movie S6), ranging from highly synchronized to completely asynchronized (Fig. 4, F, H, and I). Every neuron that fused with glial cells presented a complete loss in neuronal activity (fig. S12, E to G). Regardless of the synchronization of fused neurons, the frequency of neuronal activity was not altered (fig. S13). To further investigate the mechanisms responsible for the simultaneous firing of fused neurons, we analyzed the Ca^2+^ levels along the neuronal bridge formed between these neurons (fig. S14, A and B, and movie S7). The intracellular Ca^2+^ concentration increased within the bridges (fig. S14, C to E), forming Ca^2+^ peaks that reflected the patterns of neuronal activity (fig. S14, F and G).

## DISCUSSION

Neuroinfectious diseases transmitted by viruses represent an emerging public health threat due to the increasing appearance of new zoonotic neurotropic viruses (*47*). Viral infections of the nervous system cause a broad spectrum of acute neurological symptoms, including meningitis, encephalitis, meningoencephalitis, paralysis, and stroke, with long-term neurological sequelae or even fatal outcomes commonly observed in severe situations. Respiratory viruses such as the human respiratory syncytial virus, the influenza virus, the emerging coronaviruses (CoV), and the human metapneumovirus, among others, are now also included in the list of potential neurotropic viruses (*48*). Long-term and chronic neurological manifestations derived from viral neuroinfections are gaining increased attention, especially after the coronavirus disease 2019 (COVID-19) pandemic where long COVID has recently been termed to describe a multitude of symptoms of neurological nature that persist for months after the infection (*17*) and affect millions of people worldwide (*49*). To date, the pathophysiological mechanisms associated with the neurological symptoms derived from viral infections are only started to be elucidated, with fusion being a possible mechanism of transmission and spreading (*50*–*56*). Our results indicate that viral infections, driving the expression of viral fusogens, can initiate the irreversible fusion of brain cells, causing alteration in neuronal communication and revealing a possible pathomechanism of neuronal malfunction caused by infection. The impact on neuronal fusion will depend on the viral load in the brain and the specific areas infected; for example, in the case of SARS-CoV-2, neuronal fusion depends on the expression of hACE2 and, potentially, other accessory entry factors such as TMPRSS2 and NRP1 in neighboring neurons. Our results also imply that other viral infections can potentially cause neuronal fusion. Several viruses can cause severe neurological symptoms and/or death, such as HIV, rabies virus, Japanese encephalitis virus, vesicular stomatitis virus, poliovirus, measles virus, herpes simplex virus, varicella-zoster virus, Zika virus, cytomegalovirus, dengue virus, Nipah virus, and chikungunya virus, among others. Cell-to-cell contact has been shown to be involved in the spreading of HIV (*14*), measles (*51*), and SARS-CoV-2 (*57*), but viral-mediated neuronal fusion remained poorly understood.

Fused neurons can result in compromised neuronal circuitry and altered animal behavior, as previously shown for *C. elegans* chemosensory neurons that ectopically express endogenous fusogens (*46*). Our results demonstrate that neurons infected by viruses or expressing their viral fusogens can acquire the ability to fuse with neighboring neurons and glial cells, both in vitro and in vivo. This, in turn, results in the sharing of large molecules and even organelles and in compromised neuronal activity. While the latter can have direct implications on brain function and animals’ behavior, sharing large molecules implies a possible mechanism for the spread of toxic aggregates as observed in several neurodegenerative diseases and could also represent a mechanism of viral spreading that eludes the immune system (*12*). Retroviruses induce the formation of viral cytonemes, consisting of filopodial bridges that facilitate cell-to-cell transmission (*58*). Tunneling nanotubes are similar cellular bridges that allow communication between cells (*59*) and have been reported to mediate the transport of toxic α-synuclein aggregates (*60*). Our results demonstrate the formation of neuronal bridges that can extend hundreds of micrometers, allowing the exchange of small proteins and large mitochondria between interconnected neurons. Given the diverse range of structures that can be transported through these viral fusogen-mediated cellular structures, it is tempting to speculate that infecting viruses and other toxic aggregates may also use these pathways to spread to neighboring cells. In the case of SARS-CoV-2, the potential of fusion-mediated spreading of infection would be determined by the delivery of viral fusogen to the cell surface, which we have shown to occur, and by the pattern of ACE2-expressing neurons (*22*) that are in contact with a single infected neuron, even at large distances from their respective cell somas. The neuropathological consequences of virus-induced neuronal fusion events could underlie the remarkable association between herpes simplex virus infection and Alzheimer’s disease (*4*), HIV and Parkinson’s disease (*61*), Epstein-Barr virus and multiple sclerosis (*62*), and Zika virus or Japanese encephalitis virus with epileptic seizures (*3*, *63*). An important consideration that emerges from our results is that fused neurons remain viable albeit with altered circuitry and function. This uncharacterized, difficult-to-detect event could explain some of the neurological consequences of viral infections of the nervous system.

Most of the current immunization approaches for COVID-19 are based on expressing the spike S protein in the host cells as an epitope to trigger the immune system response (*64*). These nucleic acid-based vaccines deliver the antigen encoded as mRNA, such as in the Pfizer-BioNTech BNT162b2 and the Moderna mRNA-1273 vaccines (*65*), or as adenovirus-enclosed DNA, such as in the Oxford-AstraZeneca ChAdOx1 nCoV-19/AZD1222 (*66*) and Johnson & Johnson Ad26.COV2.S (*39*) vaccines. The current versions of the Moderna, Pfizer-BioNTech, and Johnson & Johnson SARS-CoV-2 vaccines encode the full-length spike S protein with two mutations (spike S-2P) that stabilize the prefusion conformation and inactivate its fusogenicity (*39*, *64*, *67*, *68*). We used this same mutant form of spike S-2P as a negative control, demonstrating the complete lack of fusogenicity when two consecutive prolines were added at positions 986 and 987. However, our findings demonstrate that it will be critical to consider the fusogenic potential when designing any future vaccines in which viral fusogens are to be expressed in mammalian cells.

## MATERIALS AND METHODS

### Molecular biology

Standard molecular biology methods were used. The *p15* DNA sequence was obtained from the National Center for Biotechnology Information (www.ncbi.nlm.nih.gov/gene/). The plasmid was then designed using the software “A Plasmid Editor,” and the insert was generated by Integrated DNA Technologies. The Pmec-4::p15 plasmid was constructed by subcloning *p15* between Msc I and Nhe I. The CMV::p15 plasmid was generated by subcloning *p15* into the pmaxCloning vector (Lonza, no. VDC-1040) between Hind III and Not I. The CMV::p15Δ21/22 plasmid was generated as previously described (*36*) by deletion of the amino acids 21 and 22 of the N terminus of the transmembrane domain. The CMV::SARS-CoV-2–S-2P plasmid was generated by introducing two prolines at the 986 (K986P) and the 987 (V987P) positions of the *SARS-CoV-2–S* gene of the pCMV14-3X-Flag–SARS-CoV-2 S plasmid. Mutations were generated using the QuikChange II Site-Directed Mutagenesis Kit (p15Δ21/22 forward primer, 5′-CCACCGCCAAATGCTTTTGTTGAAAGCAGTTCTACTG-3′; p15Δ21/22 reverse, primer 5′-CAGTAGAACTGCTTTCAACAAAAGCATTTGGCGGTGG-3′; SARS-CoV-2–S-2P forward, plasmid 5′-CCTGAGTCGCCTTGATCCGCCGGAAGCTGAAGTTC-3′; and SARS-CoV-2–S-2P reverse plasmid, 5′-GAACTTCAGCTTCCGGCGGATCAAGGCGACTCAGG-3′). Positive clones were confirmed by Sanger sequencing. The Kaede-N1 plasmid was a gift from M. Davidson (Addgene, plasmid no. 54726; http://n2t.net/addgene:54726; RRID:Addgene_54726) (*69*). The mito-mPA-GFP plasmid was a gift from R. Youle (Addgene, plasmid no. 23348; http://n2t.net/addgene:23348; RRID:Addgene_23348) (*70*). The mito-7-mPA-mCherry1 plasmid was a gift from M. Davidson (Addgene, plasmid no. 57189; http://n2t.net/addgene:57189; RRID:Addgene_57189) (*71*). The pCMV14-3X-Flag-SARS-CoV-2 S plasmid was a gift from Z. Qian (Addgene, plasmid no. 145780; http://n2t.net/addgene:145780; RRID:Addgene_145780) (*37*). The pcDNA3.1-SARS2-Spike plasmid was a gift from F. Li (Addgene, plasmid no. 145032; http://n2t.net/addgene:145032; RRID:Addgene_145032) (*72*). The SARS-CoV-2 S-6P plasmid was a gift from J. McLellan (Addgene, plasmid no. 154754; http://n2t.net/addgene:154754; RRID:Addgene_154754) (*40*). The pcDNA3.1-hACE2 plasmid was a gift from F. Li (Addgene, plasmid no. 145033; http://n2t.net/addgene:145033; RRID:Addgene_145033) (*72*).

### Animal ethics and mouse strains

All experimental procedures using animals were conducted under the guidelines of the Australian Code of Practice for the Care and Use of Animals for Scientific purposes and were approved by the University of Queensland Animal Ethics Committee (2019/AE000243) or the Macquarie University Animal Ethics Committee (2021/018). WT (C57BL/6 background) mice were maintained on a 12-hour light/12-hour dark cycle and housed in a PC2 facility with ad libitum access to food and water.

### Culture of cell lines

Human embryonic kidney (HEK) 293T cells (American Type Culture Collection; 293T/17, ATCCCRL-11268) were cultured in a humidified atmosphere at 37°C with 5% CO_2_ and maintained in Dulbecco’s modified Eagle’s medium (DMEM; Gibco–Thermo Fisher Scientific) supplemented with 10% fetal bovine serum (Gibco–Thermo Fisher Scientific), 1× GlutaMAX (Gibco–Thermo Fisher Scientific), and penicillin (100 U/ml)–streptomycin (100 μg/ml; Sigma-Aldrich–Merck). Vero E6 cells expressing TMPRSS2 were cultured in the same medium as the HEK-293T cells supplemented with puromycin (30 μg/ml; Sigma-Aldrich–Merck). Cells were transfected using the Lipofectamine LTX reagent according to the manufacturer’s instructions (Invitrogen–Thermo Fisher Scientific).

### Murine neuronal culture

Primary hippocampal neurons were obtained from mice at embryonic day 16. Isolated hippocampi were prepared as previously described (*73*, *74*). Briefly, 250,000 neurons were plated onto poly-l-lysine–coated 35-mm glass-bottom dishes (In Vitro Scientific) in Neurobasal medium (Gibco) supplemented with 5% fetal bovine serum (HyClone), 2% B-27, 2 mM GlutaMAX, and penicillin/streptomycin (50 U/ml; Invitrogen). The medium was changed to serum-free/antibiotic-free medium 24 hours after seeding, and half the medium was changed every week.

### hESC-derived neurons

hESC-derived cortical neurons were differentiated from hESCs (H9, WIC-WA09-MB-001; WiCell) using a modification of a previously published protocol (*75*). Briefly, the hESCs were maintained on Matrigel (#354230; Corning) in Essential 8 medium (#A1517001; Life Technologies). hESCs were dissociated with Accutase (#AT-104-500; Innovate Cell Technologies) at 37°C for 1 min and seeded on AggreWell 800 (#34815; STEMCELL Technologies) in Essential 8 medium with ROCK inhibitor Y-27632 (10 μM, #72308, STEMCELL Technologies). After 24 hours, spheroids were collected and transferred into ultralow-attachment plates (#CLS3471; Sigma-Aldrich) with Essential 6 medium (#A1516401; Life Technologies) containing dorsomorphin (2.5 μM; #P5499, Sigma-Aldrich), SB-431542 (10 μM; #1614, Tocris) and cultured until day 13. The spheroids were then collected and placed into Matrigel (Corning)–coated six-well plates with DMEM/F12 (#11330057; Thermo Fisher Scientific), supplemented with 1× N2 (#17502048; Thermo Fisher Scientific). The resultant neural progenitor cells were passaged until day 30 and then replated on poly-l-lysine–coated (10 μg/ml; #P2636; Sigma-Aldrich) and laminin-coated (20 μg/ml; #23017015; Thermo Fisher Scientific) plates and maintained in Neurobasal medium (#21103049; Thermo Fisher Scientific) supplemented with B-27 (Thermo Fisher Scientific), GlutaMAX (#35050061; Thermo Fisher Scientific), brain-derived neurotrophic factor (BDNF; #78133; STEMCELL Technologies), ascorbic acid (200 μM; #72132; STEMCELL Technologies), GDNF (20 ng/ml; #78139.1, STEMCELL Technologies), and cyclic adenosine monophosphate (1 μM; #A6885; Sigma-Aldrich) up to 50 days.

### Brain organoids

hESC-derived brain organoids were produced as previously reported (*76*). Briefly, hESCs were incubated with Accutase (#AT-104-500; Innovate Cell Technologies) at 37°C for 1 min. Dissociated single cells were seeded on AggreWell 800 (#34815, STEMCELL Technologies). One thousand single cells were added per well in Essential 8 medium supplemented with 10 μM ROCK inhibitor Y-27632 and centrifuged at 100*g* for 3 min. The AggreWell 800 plates were incubated at 37°C and 5% CO_2_. After 24 hours, the organoids were transferred into ultralow-attachment 60ɸ dishes (#CLS3261, Sigma-Aldrich) in Essential 6 medium supplemented with 2.5 μM dorsomorphin, 10 μM SB-431542, and 2.5 μM XAV-939 (#3748, Tocris). The medium was changed every day for 5 days. On day 6, the medium was changed into Neurobasal A (#10888; Thermo Fisher Scientific) containing GlutaMAX (#35050; Thermo Fisher Scientific) and B-27 without vitamin A (#12587; Thermo Fisher Scientific). The medium was supplemented with epidermal growth factor (20 ng/ml; #236-EG; R&D Systems) and basic fibroblast growth factor (20 ng/ml; #233-FB; R&D Systems) until day 24, when it was switched to Neurobasal A medium containing GlutaMAX and B-27 without vitamin A and supplemented with BDNF (20 ng/ml) and neurotrophin-3 (20 ng/ml; #78074; STEMCELL Technologies) until day 43. Last, the medium was changed to Neurobasal A containing GlutaMAX and B-27 without vitamin A. For the viral labeling of brain organoid neurons, the adenovirus (AAV) *hSyn-eGFP* were constructed as previously described (*77*). Viral transduction was performed on 43 to 50 DIV organoids in ultralow-attachment 96-well plates (Corning) using 5 × 10^11^ titers of virus and exchanging the medium after 3 days. Transduced organoids were maintained for 10 additional days before SARS-CoV-2 infection.

### SARS-CoV-2 preparation and infection

SARS-CoV-2 isolate hCoV-19/Australia/QLD02/2020 (QLD02) (used as the original ancestral virus) was provided by Queensland Health Forensic and Scientific Services, Queensland Department of Health. The virus was amplified in Vero cells expressing human TMPRSS2 (hTMPRSS2) and titrated by plaque assay. To evaluate the neuronal fusogenicity of SARS-CoV-2, 2D neuronal cultures were inoculated with 2 × 10^5^, 2 × 10^3^, or 20 PFUs or were mock-infected with control culture media, resuspended in neuronal culture medium, and incubated at 37°C until fixation (30 min) with paraformaldehyde [4% in phosphate-buffered saline (PBS)] 72 hours later. 3D hESC-derived brain organoids were inoculated with 2 × 10^4^ PFUs or were mock-infected, all resuspended in brain organoid culture medium and incubated at 37°C until fixation (30 min) with paraformaldehyde (4% in PBS) 72 hours later. All experiments involving live SARS-CoV-2 and SARS-CoV followed the approved standard operating procedures in the Biosafety Level 3 facility from the School of Chemistry and Molecular Biosciences at the Faculty of Science of The University of Queensland.

### Electroporation and transfection

When required, murine neurons were electroporated using the Invitrogen Neon transfection system (#MPK1025, Thermo Fisher Scientific) following the manufacturer’s instructions. Briefly, immediately after dissection, isolated neurons were washed twice in Ca^2+^-Mg^2+^–free PBS and resuspended in buffer R containing 1 to 2 μg of the DNA to electroporate. The conditions for the electroporation were as follows: voltage, 1500 V; width, 10 ms; and three pulses. Alternatively, the neurons were transfected after 7 to 12 DIV using the Lipofectamine 2000 (#11668027, Invitrogen) reagent as previously described (*73*). Following the manufacturer’s instructions, human neurons were transfected with Lipofectamine 3000 (#L3000015, Thermo Fisher Scientific) and 1.6 μg of plasmid DNA at 40 to 50 DIV; human brain organoids were transfected with Lipofectamine 3000 and 2.4 μg of plasmid DNA at 43 to 50 DIV. The transfection medium was replaced after 24 hours.

### *C. elegans* strain maintenance, genetic crosses, and manipulation

Standard techniques were used for *C. elegans* maintenance, genetic crosses, and manipulations (*78*). Experiments were performed on hermaphrodite animals grown at room temperature (~22°C) on nematode growth medium plates, seeded with OP50 *Escherichia coli*. Transgenic strains generated during this study were obtained by standard microinjection techniques (*79*) and include *vdEx1266* [*Pmec-4::p15*; 5 ng/μl], *vdEx1268* [*Pmec-4::p15*; 5 ng/μl]; *vdEx1417* [*Pmec-4::p15*Δ*21/22*; 15 ng/μl], *vdEx1487* [*Pmec-4::p15Δ21/22*; 5 ng/μl], and *vdEx1489* [*Pmec-4::p15Δ21/22*; 5 ng/μl]. All injection mixes had a total concentration of 100 ng/μl and contained the transgene of interest, empty pSM plasmid as a filler, and a co-injection marker for the identification of transgenic animals. As the cell cycle transfer of extrachromosomal arrays (*vdEx*) is unstable, some animals lose the transgene. This provides an internal control for each transgenic strain, with these controls being referred to as “nontransgenic siblings” or *transgene (−)*.

### Murine brain AAV transduction and brain histochemistry

AAV expressing either e*GFP* alone (control) or e*GFP* and the viral fusogen *p15* under the *hSyn* promoter were stereotactically injected at the level of the cortex (2 mm posterior to bregma, 2 mm bilateral to midline, and 0.8 mm deep) and the hippocampus (2 mm posterior to bregma, 2 mm lateral to midline, and 2 mm deep) of adult WT mice (11 weeks old). Control and p15-injected animals were randomly divided into two groups based on the duration of the transduction, and brains were processed either 7 or 14 days after infection. Briefly, mice were anesthetized and transcardially perfused with cold PBS. Brains were then removed, post-fixed with 4% (w/v) paraformaldehyde (PFA) in PBS overnight, and cryoprotected with 30% (w/v) sucrose in PBS overnight. Frozen brains were cryosectioned at 80 μm; they were permeabilized with 0.5% (v/v) Triton X-100 (#X100; Sigma-Aldrich) in PBS for 30 min and blocked in 5% (w/v) BSA (#A7906; Sigma-Aldrich), 1% horse serum (#26050070; Thermo Fisher Scientific), and 0.1% (v/v) Triton X-100 overnight. The brains were then incubated with primary antibodies to GFP (#ab290; Abcam) and NeuN (#ab091; Merck/MilliporeSigma) at 4°C for 3 days. Following PBS washing, they were incubated at 4°C for 2 days with Alexa Fluor–conjugated secondary antibodies (#A32732 or #A-11039; Thermo Fisher Scientific), and nuclei were counterstained with DAPI (4′,6-diamidino-2-phenylindole; #D1306; Invitrogen) before being washed with PBS and prepared for confocal imaging.

### Immunofluorescence staining

Murine neurons and cells were fixed with a solution of 4% PFA in PBS for 10 min. Neurons were rinsed in PBS and permeabilized with a solution of 0.1% Triton-X-100 in PBS for 10 min. They were then washed with PBS and incubated in blocking solution (5% horse serum and 1% BSA in PBS) for 1 hour at room temperature. After blocking, the neurons were incubated with the primary antibodies to GFP (#AB16901, Merck Millipore), mCherry (#ab167453, Abcam), NRP1 (#ABIN1173423, Antibodies-online.com), TMPRSS2 (#ab109131, Abcam), and MAP2 (#188004, Synaptic Systems), diluted in primary antibody solution (1% BSA in PBS) overnight at 4°C. The antibody anti-TMPRSS2 required an antigen retrieval step performed after fixation. After incubation with the primary antibodies, the cells were then washed with PBS and incubated with the secondary antibodies Alexa Fluor 488 goat anti-chicken (#A-11039, Thermo Fisher Scientific), Alexa Fluor 555 goat anti-rabbit (#A32732, Thermo Fisher Scientific), Alexa Fluor 647 goat anti-guinea pig (#A21450, Thermo Fisher Scientific), and DAPI (#62248, Thermo Fisher Scientific) in the secondary antibody solution (5% horse serum in PBS) for 1 hour at room temperature. Last, the neurons were washed and mounted in VECTASHIELD PLUS Antifade mounting medium (#H-2000, Vector Laboratories). The staining protocol was slightly modified for human neurons; they were fixed for 5 min, permeabilized with 0.1% (v/v) Triton-X-100 for 10 min, blocked with 5% (w/v) BSA (#A9418, Sigma-Aldrich), and stained for MAP2 only. Organoids were fixed with 4% PFA for 30 min at room temperature. PBS-rinsed organoids were permeabilized with 0.5% (v/v) Triton X-100 (#X100; Sigma-Aldrich) in PBS for 30 min and blocked in 5% (w/v) BSA for 5 hours. The organoids were then incubated with primary antibodies to MAP2 (#ab5392, Abcam) at 4°C for 3 days. Following PBS washing, they were incubated with Alexa Fluor–conjugated secondary antibodies (#A11039 or #A21437, Thermo Fisher Scientific) at 4°C for 3 days. Nuclei were counterstained with DAPI for 30 min and washed with PBS before imaging.

### Confocal microscopy of fixed samples

Immunofluorescence imaging was carried out using a Zeiss Plan Apochromat 40×/1.2 numerical aperture (NA) water-immersion objective on a confocal/two-photon laser scanning microscope (LSM 710; Carl Zeiss) built around an Axio Observer Z1 body (Carl Zeiss), equipped with two internal gallium arsenide phosphide (GaAsP) photomultiplier tubes (PMTs) and three normal PMTs for epi (descanned) detection, or using a confocal microscope (LSM 880, Carl Zeiss) built around an Axio Observer Z1 body (Carl Zeiss), equipped with two internal gallium arsenide phosphide (GaAsP) PMTs and three normal PMTs for epi (descanned) detection. Both systems were controlled by Zeiss Zen Black software. Images were further processed and analyzed with Fiji-ImageJ (*80*). Imaging of organoids and mice brain slices was performed using a Yokogawa W1 inverted spinning disc confocal controlled by SlideBook 6.0 software (3I Inc.). Large-field imaging of tissue sections was performed using a 20×/0.8 NA air objective and a Hamamatsu Flash4.0 scientific complementary metal-oxide semiconductor (sCMOS) camera. High-resolution *Z*-stacks were performed using a 63×/1.4 NA oil-immersion objective and a Photometrics Evolve electron-multiplying charge-coupled device (EMCCD) camera.

### Confocal and epifluorescence live imaging

*C. elegans* animals were immobilized using 0.05% tetramisole hydrochloride on 3.5% agar pads. The animals were imaged with a Zeiss Axio Imager Z1 microscope equipped with a Photometrics camera (Cool Snap HQ2) and analyzed using MetaMorph software (Molecular Devices) and Fiji-ImageJ. Cytoplasmic GFP was visualized with 470/80-nm excitation and 525/50-nm emission filters, and mitochondria tagged with a monomeric red fluorescent protein (mRFP) were visualized with 545/25-nm excitation and 605/70-nm emission filters. Animals were imaged on a spinning-disk confocal system (Marianas; 3I Inc.) consisting of an Axio Observer Z1 (Carl Zeiss) equipped with a CSU-W1 spinning-disk head (Yokogawa Corporation of America), an ORCA-Flash4.0 v2 sCMOS camera (Hamamatsu Photonics), and a 100×/1.4 NA Plan-Apochromat objective. Image acquisition was performed using SlideBook 6.0 and processed using Fiji-ImageJ.

A photoactivation assay was performed to demonstrate the exchange of mitochondria between fused mammalian neurons. For live-cell imaging of mito-mPA-GFP diffusion, a UV pulse was applied on a 10 μm–by–10 μm region of interest (ROI). Before photoactivation, mito-mPA-GFP is not visible as it remains caged (nonfluorescent). After irradiation with UV light, mPA-GFP irreversibly photoconverts to a green-emitting fluorescent protein. Photoconversion resulted in the emergence of green mitochondria that slowly moved from one neuron to the other along the fusion bridge. Green images were collected every 5 min; seventy-two images were acquired after photoconversion, and one last image was acquired 13 hours later. To identify a bidirectional transfer of mitochondria between fused neurons, 5 DIV neurons expressing either spike S, blue fluorescent protein (BFP), and mito-mPA-mCherry 1 or hACE2, BFP, and mito-mPA-GFP were imaged. Fused BFP-positive neuronal syncytia were identified by using a 405-nm excitation laser. This identification step also photoconverted both mito-mPA-GFP and mito-mPA-mCherry 1, making them visible and allowing the identification of interchanged mitochondria between fused neurons.

Fluorescence photoconversion was used to show the diffusion of large molecules between fused mammalian neurons. For live-cell imaging of Kaede diffusion, fusogen/empty vector-Kaede–transfected neurons were bathed in imaging buffer [145 mM NaCl, 5.6 mM KCl, 2.2 mM CaCl_2_, 0.5 mM MgCl_2_, 5.6 mM d-glucose, 0.5 mM ascorbic acid, 0.1% BSA, and 15 mM Hepes (pH 7.4)]. Neurons were visualized at 37°C on a Zeiss Plan Apochromat 40x/1.2 NA water-immersion objective on a confocal/two-photon laser scanning microscope (LSM 710; Carl Zeiss). In transfected neurons, a UV pulse was applied on a 5 μm–by–5 μm ROI. Photoconversion resulted in the emergence of a spot of red fluorescence that rapidly diffused through the soma and proximal neurites. Simultaneous green and red images were collected every 785 ms; five images were acquired before photoconversion, and 50 images were acquired after photoconversion. Photoconversion and diffusion of the fluorophore were performed on neurons separated by 25 to 100 μm. In control conditions, UV light was applied first outside the neuron, 50 μm away from the soma, and then inside the soma.

Ca^2+^ imaging to visualize spontaneous neuronal activity was performed on differentiated neuronal primary cultures (14 to 18 DIV). Neurons were transfected with mCherry together with either the control empty vector or p15 and imaged 3 days later. Neurons were incubated at 37°C for 2 hours in Neurobasal medium supplemented with 2 mM GlutaMAX, 2% B-27, 10 μM Cal-520 AM (Abcam), and 0.03% Pluronic F-127 (Sigma-Aldrich). Following incubation, neuronal cultures were washed three times, transferred to imaging buffer, and imaged at 37°C on a Zeiss Plan Apochromat 40×/1.2 NA water-immersion objective on a confocal/two-photon laser scanning microscope (LSM 710; Carl Zeiss).

### Neuron-neuron, neuron-glia, and glia-glia fusion quantification in mammalian neuronal cultures, human-derived brain organoids, and murine brain sections

Cell-cell fusion efficiency in neuronal cultures was quantified as the percentage of transfected neurons (GFP or mCherry) with their somas within a radius ≤200 μm and that contained simultaneously both GFP and mCherry. Neuron-neuron fusion was identified as more than two fused cells that were both positive for MAP2 staining. Neuron-glia fusion was identified by one of the fused cells being positive for MAP2. Glia-glia fusion was identified by neither of the fused cells being positive for MAP2. Neuronal syncytia were quantified as the percentage of interconnected neurons within a distance ≤200 μm. Neuron-neuron fusion in human-derived brain organoids and in murine brain sections was quantified as the percentage of pairs of transfected neurons that appear as tightly interconnected, with no gaps between them. In brain tissues, neuronal fusion was inspected within voxels of 0.01236 mm^3^. Over 100 voxels through each brain region (hippocampus or cortex) were analyzed per animal. *Z*-stacks of images were deconvolved using Huygens software (Scientific Volume Imaging), and the 3D analysis of neuronal fusion was performed using the 3D viewer plugin from Fiji-ImageJ.

### Statistical analysis

Results were analyzed statistically using GraphPad Prism software (GraphPad Software Inc.). The D’Agostino and Pearson test was used to test for normality. The unpaired two-tailed nonparametric Mann-Whitney *U* test was used for comparison of two groups, when the data were not normally distributed. The unpaired two-tailed Welch’s *t* test was used for comparison of two groups, when the data were distributed following normality. For datasets comparing more than two groups, we performed one-way analysis of variance (ANOVA) Kruskal-Wallis test followed by Dunn’s post hoc test for multiple comparisons, one-way ANOVA Brown-Forsythe and Welch tests followed by the Games-Howell’s post hoc test for multiple comparisons, or two-way ANOVA followed by Geisser-Greenhouse correction and the Šidák post hoc test for multiple comparisons. Statistical comparisons were performed on a per-dish, per-neuron, per–human-derived organoid, or per-animal basis, depending on the experiment, as stated in the figure legends. Two to four technical replicate dishes were imaged, and two to five independent cultures were used per condition. One hundred to 103 *C. elegans* nematodes, three mice brains, and three to eight human-derived organoids were used per condition, as stated in the figure legends. Each mouse dissection provided neurons from a pool of at least six different embryos. Values are represented as means ± SD or means ± SEM. The tests used are indicated in the respective figure legends. A *P* value below 0.05 was accepted as significant.

## Notes

View/request a protocol for this paper from *Bio-protocol*.
