# Supplementary material for: SARS-CoV-2 infection and viral fusogens cause neuronal and glial fusion that compromises neuronal activity
Source: Sci Adv. 2023 Jun 7;9(23):eadg2248. doi: 10.1126/sciadv.adg2248 (PMC10246911; doi:10.1126/sciadv.adg2248)

Science Advances
AAAS

Supplementary Materials for

SARS-CoV-2 infection and viral fusogens cause neuronal and glial fusion that compromises neuronal activity

Ramón Martínez-Mármol *et al.*

Corresponding author: Massimo A. Hilliard, m.hilliard@uq.edu.au

Sci. Adv. **9**, eadg2248 (2023)
DOI: 10.1126/sciadv.adg2248

The PDF file includes:

Figs. S1 to S14
Legends for movies S1 to S7

Other Supplementary Material for this manuscript includes the following:

Movies S1 to S7

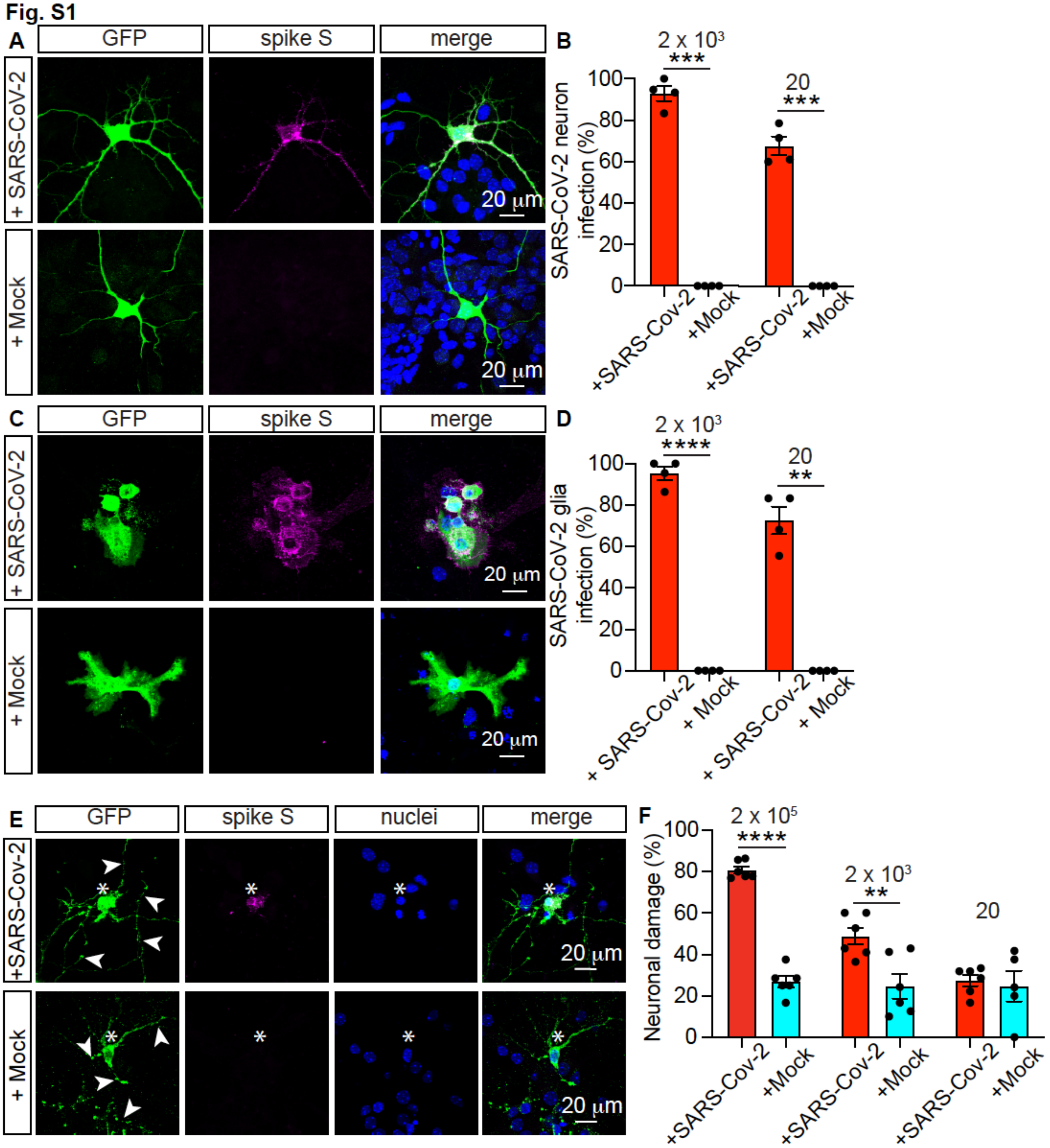

Fig. S1.

SARS-CoV-2 infects neurons and glial cells.

(**A**), Representative images of SARS-CoV-2-infected neurons (upper row) identifiable as being positive for GFP (green) and spike-S protein (magenta), and non-fused mock-control-infected neurons (lower rows), which are positive for GFP (green) but negative for spike-S protein. (**B**), Quantification of neuronal SARS-CoV-2 infection in the murine hippocampal cultures, measured as the percentage of GFP neurons that were also positive for spike S protein. (**C**), Representative images of SARS-CoV-2-infected glial cells (upper row) identifiable as being positive for GFP

(green) and spike-S protein (magenta), and non-fused mock-control-infected glial cells (lower row), which are positive for GFP (green) but negative for spike-S protein. (**D**), Quantification of glial SARS-CoV-2 infection in the murine hippocampal cultures, measured as the percentage of GFP glial cells that were also positive for spike S protein. (**E**), Representative images showing that a high dose of SARS-CoV-2 infection is associated with neuronal damage. SARS-CoV-2-infected neurons (upper row) were identifiable as being positive for GFP (green) and spike S (magenta), and non-infected mock-treated neurons identifiable as positive for GFP (green) but negative for spike S (magenta), with signs of neuronal damage (arrowheads). (**F**), Quantification of the percentage of fluorescent neurons with signs of neuronal damage. Data in (B), (D) and (F) are displayed as mean ± SEM. n = 4 neuronal cultures from 2 independent infections > 100 neurons were counted per dish in (B) and (D). n = 6 dishes from 3 neuronal cultures and 3 independent infections in (F). 15 to 41 cells were counted per dish. Unpaired two-tailed Welch's *t* test were used in (B), (D) and (F). $^{**}p < 0.01$, $^{***}p \leq 0.001$, $^{****}p \leq 0.0001$.

Fig. S2

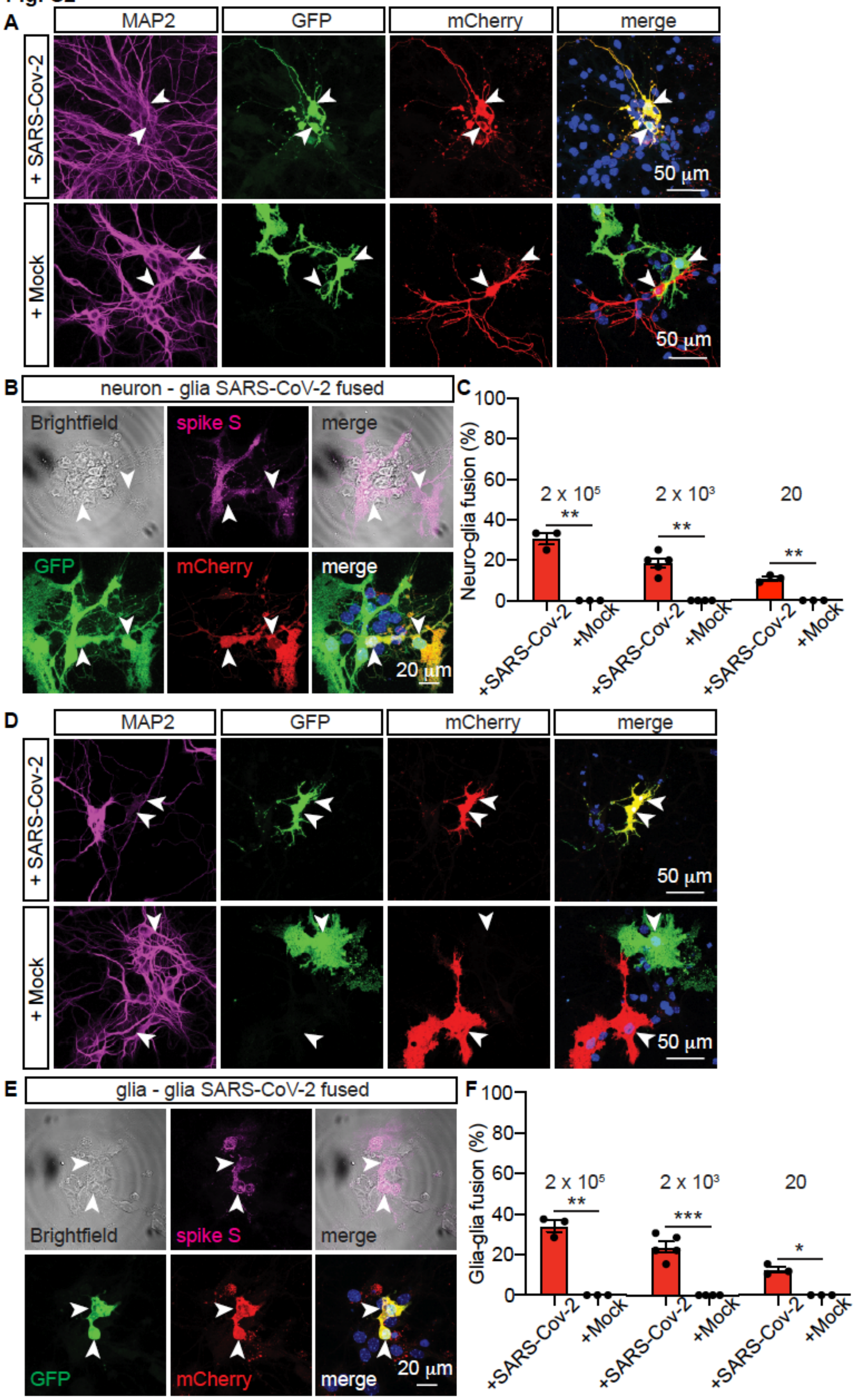
A
MAP2
GFP
mCherry
merge
+ SARS-Cov-2
+ Mock
50 μm
50 μm
B
neuron - glia SARS-CoV-2 fused
Brightfield
spike S
merge
GFP
mCherry
merge
20 μm
C
Neuro-glia fusion (%)
100
80
60
40
20
0
2 x 10⁵
2 x 10³
20
**
**
**
+SARS-Cov-2
+Mock
D
MAP2
GFP
mCherry
merge
+ SARS-Cov-2
+ Mock
50 μm
50 μm
E
glia - glia SARS-CoV-2 fused
Brightfield
spike S
merge
GFP
mCherry
merge
20 μm
F
Glia-glia fusion (%)
100
80
60
40
20
0
2 x 10⁵
2 x 10³
20
**
***
*
+SARS-Cov-2
+Mock

Fig. S2.

SARS-CoV-2 infection induces neuron-glial and glial-glial fusion.

(**A**), Representative images of neuro-glia fusion upon SARS-CoV-2 infection (upper row) identifiable by GFP (green) and mCherry (red) fluorescence appearing simultaneously in an adjacent neuron and glial cells (yellow in the merged panel). Non-fused mock-control-infected neurons and glial cells (lower row) were identified as adjacent green or red neurons and glial cells. (**B**), Representative images of neuron-glia fused cells (arrowheads) positive for spike S staining. (**C**), Quantification of neuro-glial fusion as the percentage of fluorescent neurons fused to glial cells (seen as yellow) from the total population of neurons and glial cells expressing GFP or mCherry. (**D**), Representative images of fused glial cells upon SARS-CoV-2 infection (upper row) identifiable by GFP (green) and mCherry (red) fluorescence appearing simultaneously in adjacent glial cells (yellow in the merged panel). Non-fused mock-control-infected glial cells (lower row) were identified as adjacent green or red glial cells. (**E**), Representative images of fused glial cells (arrowheads) positive for spike S staining. (**F**), Quantification of glia-glia fusion as the percentage of fused fluorescent glial cells (seen as yellow) from the total population of glial cells expressing GFP or mCherry. Images in (A) and (D) show immunocytochemistry for nuclei (blue), neuronal MAP2 (magenta), GFP (green) and mCherry (red). Neurons were identified as cells positive for MAP2, and non-neuronal glial cells were defined based on their morphology and the absence of MAP2 staining. Images in (B) and (E) show immunocytochemistry for nuclei (blue), GFP (green), mCherry (red) and SARS-CoV-2 spike S (magenta). In all images, arrowheads indicate cell bodies. Data in (C) and (F) are displayed as mean ± SEM. n = 3 - 4 dishes from 3 neuronal cultures and 3 independent infections. 13 to 41 cells were counted per dish. Unpaired two-tailed Welch's *t* tests were used in (C) and (F). $*p \leq 0.05$, $**p < 0.01$, $***p \leq 0.001$.

Fig. S3

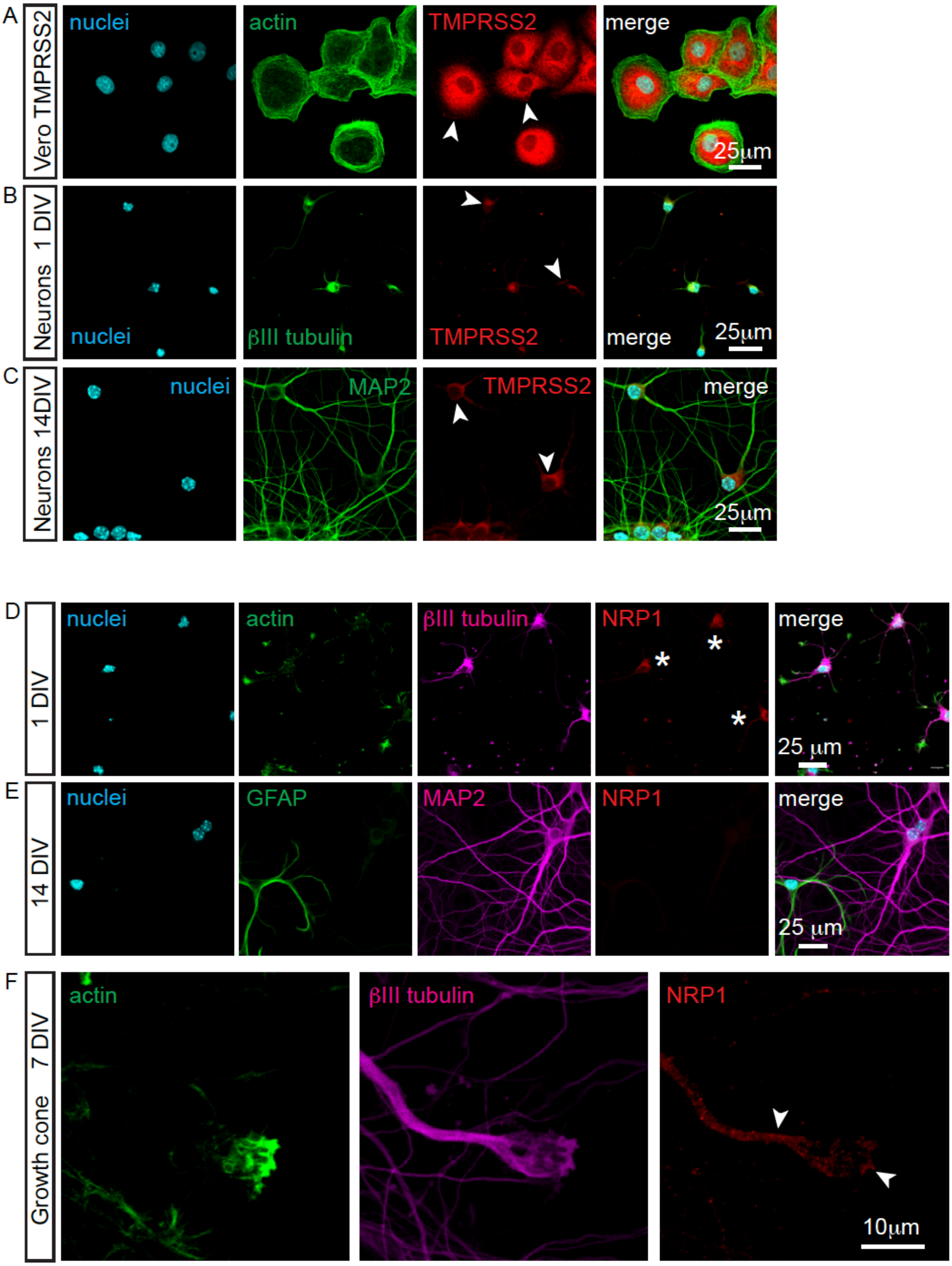

A
Vero TMPRSS2
nuclei
actin
TMPRSS2
merge
25μm
B
Neurons 1 DIV
nuclei
βIII tubulin
TMPRSS2
merge
25μm
C
Neurons 14DIV
nuclei
MAP2
TMPRSS2
merge
25μm
D
1 DIV
nuclei
actin
βIII tubulin
NRP1
merge
25 μm
E
14 DIV
nuclei
GFAP
MAP2
NRP1
merge
25 μm
F
Growth cone 7 DIV
actin
βIII tubulin
NRP1
10μm

Fig. S3.

TMPRSS2 and NPR1 are detected in neurons.

(**A**), Representative images of TMPRSS2 in African green monkey kidney epithelial Vero cells expressing human TMPRSS2. TMPRSS2 is observed intracellularly and on the surface of the cells (arrowheads). (**B**, **C**), Representative images of TMPRSS2 in hippocampal cultured mouse neurons at 1 DIV (B) and at 14 DIV (C). Low levels of TMPRSS2 were observed intracellularly and on the surface of the neurons (arrowheads). (**D**, **E**), Representative images of NRP1 in hippocampal cultured mouse neurons at 1 DIV (D); NPR1 was below detection in 14 DIV differentiated neurons (E). (**F**) Representative images of NRP1 on the surface of the axon and growth cones (arrowheads) of undifferentiated 7 DIV neurons.

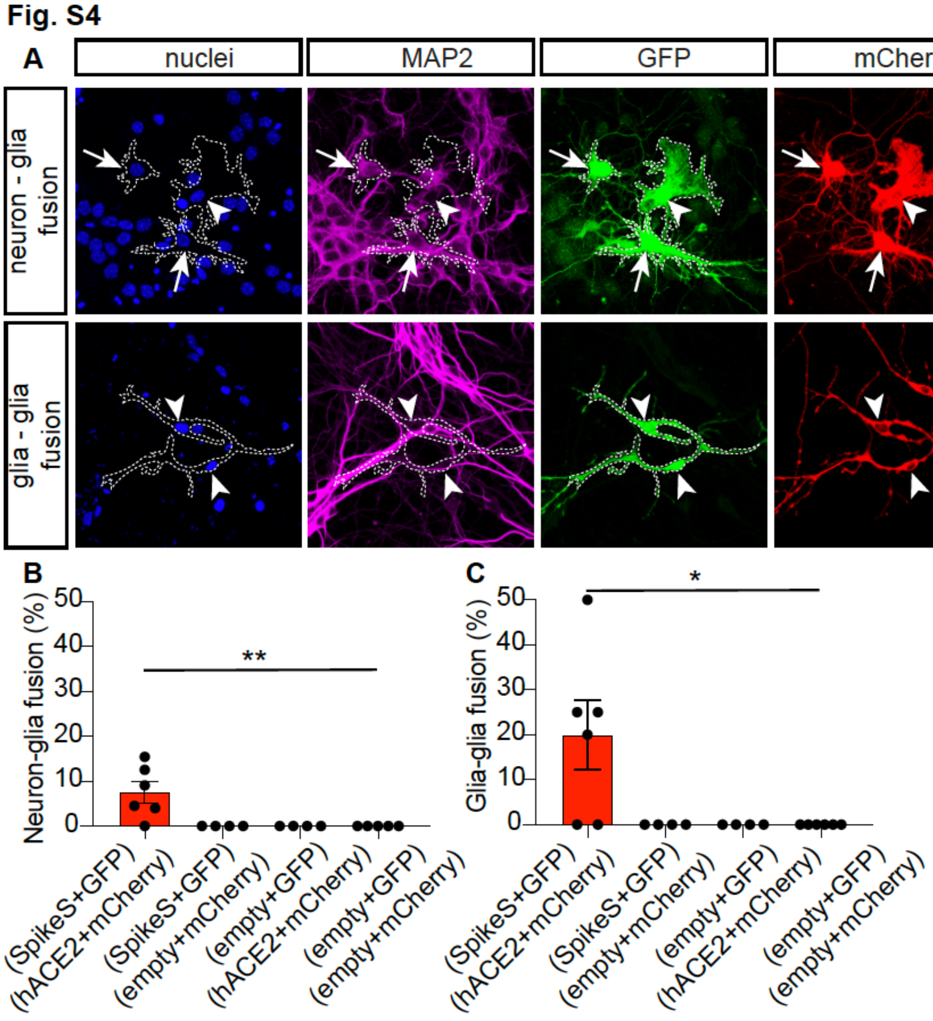

Fig. S4.

The expression of spike S and its receptor hACE2 is sufficient to induce neuron-glia and glia-glia fusion in culture.

(**A**), Representative images of fusion between neurons and glial cells (top row), or between two glial cells (bottom row). Two populations of hippocampal cells, one expressing spike S and GFP and the other hACE2 and mCherry, and then co-cultured for 7 DIV. Immunocytochemistry for nuclei (blue), MAP2 (magenta), GFP (green) and mCherry (red). Fusion is visualized by the presence of GFP and mCherry in the same cells (yellow in the merge panel). Neurons are identified as being positive for MAP2 staining (arrows in first row), whereas non-neuronal glial cells are identified as being negative for MAP2 staining (arrowheads). Fusion does not occur when the neurons are transfected with empty vector and GFP, or empty vector and mCherry. (**B**), Quantification of neuron-glial fusion as the percentage of cells that fused (yellow) when two neurons/glial cells were in close proximity (≤ 200 μm). (**C**), Quantification of non-neuronal fusion as the percentage of glial cells that fused (yellow) when two were in direct contact. Data in (B) and (C) are displayed as mean ± SEM, n > 200 brain cells analyzed in 4-6 independent dishes from 2 dissections; one-way ANOVA Kruskal-Wallis test followed by Dunn's *post hoc* test was used to compare all groups to the group without spike S or hACE2. $*p < 0.05$, $**p < 0.01$.

Fig. S5

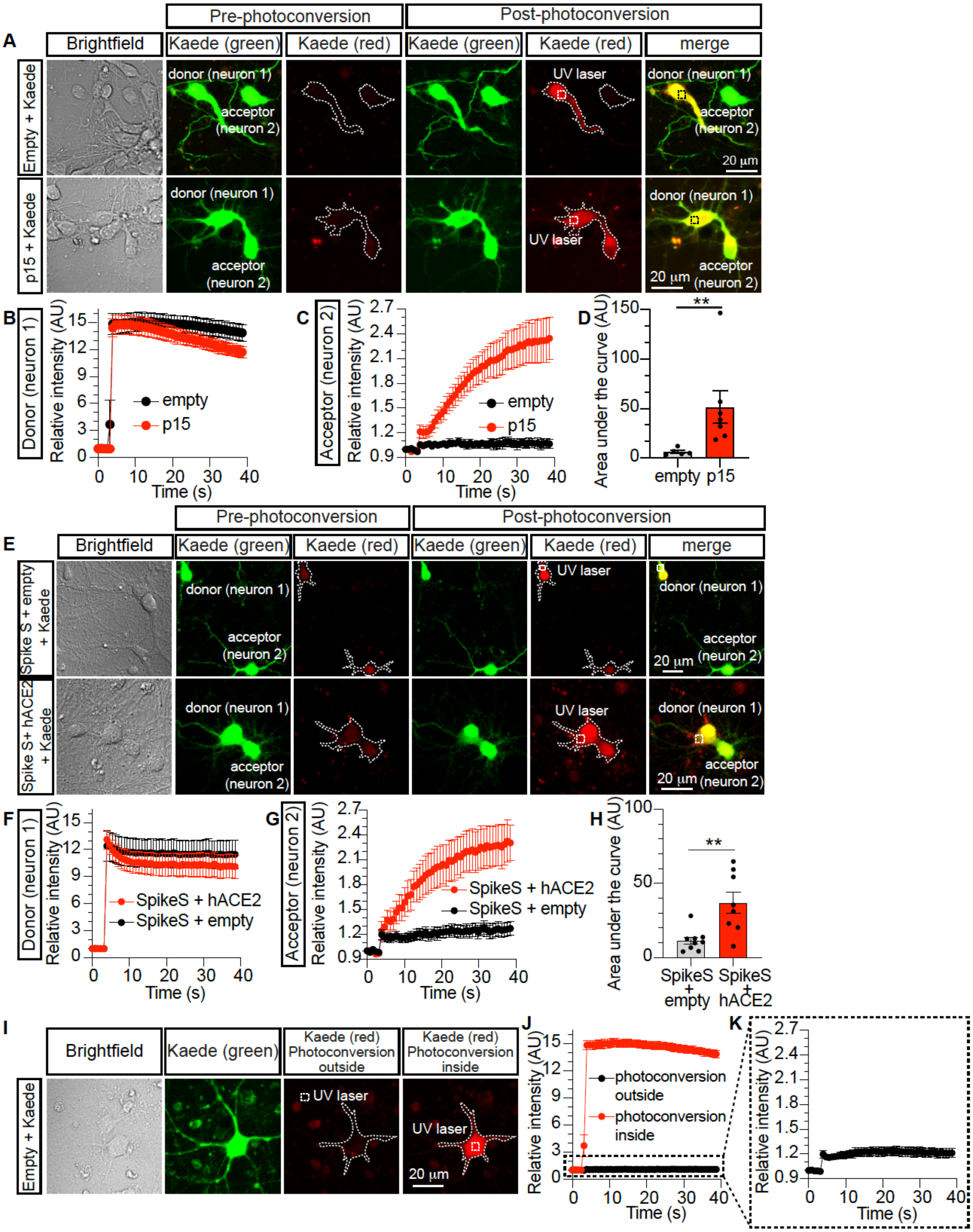
A
Pre-photoconversion
Post-photoconversion
Brightfield
Kaede (green)
Kaede (red)
merge
Empty + Kaede
p15 + Kaede
donor (neuron 1)
acceptor (neuron 2)
UV laser
20 µm
B
Donor (neuron 1)
Relative intensity (AU)
empty
p15
Time (s)
C
Acceptor (neuron 2)
D
Area under the curve (AU)
**
E
Spike S + empty + Kaede
Spike S+ hACE2 + Kaede
F
SpikeS + hACE2
SpikeS + empty
G
H
I
Kaede (red) Photoconversion outside
Kaede (red) Photoconversion inside
J
photoconversion outside
photoconversion inside
K

Fig. S5.

Diffusion of photoconvertible fluorophores between p15 and spike S-fused neurons.

(**a**), Representative images of non-fused control (empty vector) neurons (upper row), or fused (p15) neurons (lower row). Hippocampal neurons were co-transfected at 7 DIV with either empty vector and Kaede (control) or p15 and Kaede. Before photoconversion (pre-photoconversion), Kaede displays a major green fluorescence emission when excited at 488 nm and negligible red emission when excited at 561 nm. After irradiation with UV light (post-photoconversion), Kaede irreversibly photoconverts to a red-emitting fluorescent protein. In the absence of neuronal fusion (upper panels), newly photoconverted red Kaede molecules cannot diffuse between adjacent cells. However, when two neurons are fused (lower panels), newly generated red photoconverted Kaede molecules rapidly diffuse from the site of photoconversion (donor-neuron 1) to the adjacent fused neuron (acceptor-neuron 2). (**B**)**,** Quantification of the decrease in the red fluorescence within the donor neurons in the absence of fusion (empty vector) or after fusion (p15). (**C**), Quantification of the increase in red fluorescence within the acceptor neurons in the absence of fusion (empty vector) or after fusion (p15). (**D**)**,** Quantification of the area under the curve of the graph in C. (**E**), Representative images of non-fused control (spike S-empty vector) neurons (upper row), or fused (spike S-hACE2) neurons (lower row). Hippocampal neurons were co-transfected at 7 DIV with either spike S, empty vector, and Kaede (control), or spike S, hACE2 and Kaede. Before photoconversion, Kaede displays a major green fluorescence emission when excited at 488 nm and negligible red emission when excited at 561 nm. After irradiation with UV light, Kaede irreversibly photoconverts to a red-emitting fluorescent protein. In the absence of neuronal fusion (upper row), newly photoconverted red Kaede molecules could diffuse between adjacent cells. However, when two neurons are fused (lower row), newly generated red photoconverted Kaede molecules rapidly diffused from the site of photoconversion (donor-neuron 1) to the adjacent fused neuron (acceptor-neuron 2). (**F**), Quantification of the decrease in the red fluorescence within the donor neurons in the absence of fusion (spike S-empty vector) or after fusion (spike S-hACE2). (**G**), Quantification of the increase of red fluorescence within the acceptor neurons in the absence of fusion (spike S-empty vector) or after fusion (spike S-hACE2). (**H**), Quantification of the area under the curve of the graph in G. (**I**), Representative image of a control, non-fused neuron where the photoconversion was performed 50 μm outside, or within the same neuron. (**J**), Quantification of the increase of red fluorescence after outside or inside photoconversion. (**K**), Magnification of the graph obtained after outside photoconversion of control neurons. Data in (B)-(D) are displayed as mean ± SEM, n = 8 and 9 neurons from 2 independent experiments. Data in (F)-(H) are displayed as mean ± SEM, n = 5 and 7 neurons from 2 independent experiments. Mann-Whitney U test was used. $**p < 0.01$.

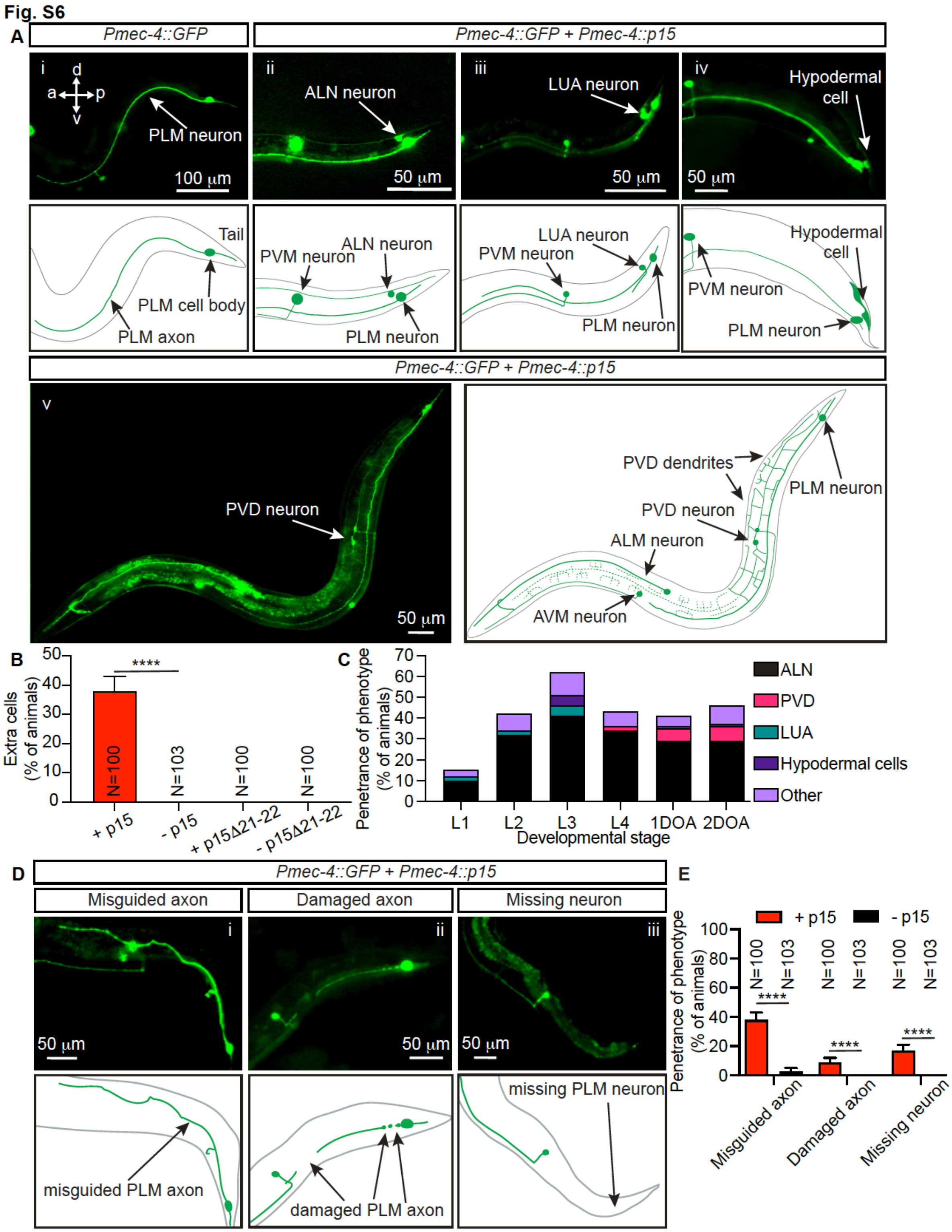

Fig. S6.

Expression of p15 induces neuronal fusion *in vivo* in *C. elegans* neurons.

(**A**), Representative images of *C. elegans* expressing GFP in the six mechanosensory neurons (*Pmec-4::GFP*) in control conditions (i.e. no fusogen present, panel i), or when co-expressing

p15 within the same neurons (*Pmec-4::GFP + Pmec-4::p15*) (panels ii, iii, iv, v). The anterior (a), posterior (p), dorsal (d) and ventral (v) axes are maintained through all the images and the focus is on the posterior lateral mechanosensory neuron (PLM). Representative images of the appearance of additional cells in animals expressing active p15: ALN neurons (ii), LUA neurons (iii), hypodermal (glial-like) cells (iv), and PVD neurons (v). (**B**), Quantification of the percentage of animals presenting extra cells in animals expressing p15 (+p15), in non-transgenic siblings (- p15), or animals expressing inactive p15 (+p15Δ21-22) and their non-transgenic siblings. (**C**), Quantification of the additional GFP-positive cells phenotype in *C. elegans* expressing p15 and GFP in mechanosensory neurons. (**D**), Representative images of *C. elegans* expressing p15 and GFP in mechanosensory neurons with PLM neurons defects in axonal guidance (first column), axonal damage (middle column) or complete neuronal absence (third column). (**E**), Quantification of the PLM neurons phenotypes in *C. elegans* expressing p15 and GFP in mechanosensory neurons. Data in (B) and (E) are displayed as mean ± SD, with the number of animals analyzed per condition (N) listed in the bar graph. One-way ANOVA Brown-Forsythe followed by Games-Howell's *post hoc* test in (B), and unpaired two-tailed Welch *t* tests in (E), comparing each mutant with its non-transgenic siblings (- transgene). 97 to 115 animals were analyzed per phenotype in (C). *****p* <0.0001.

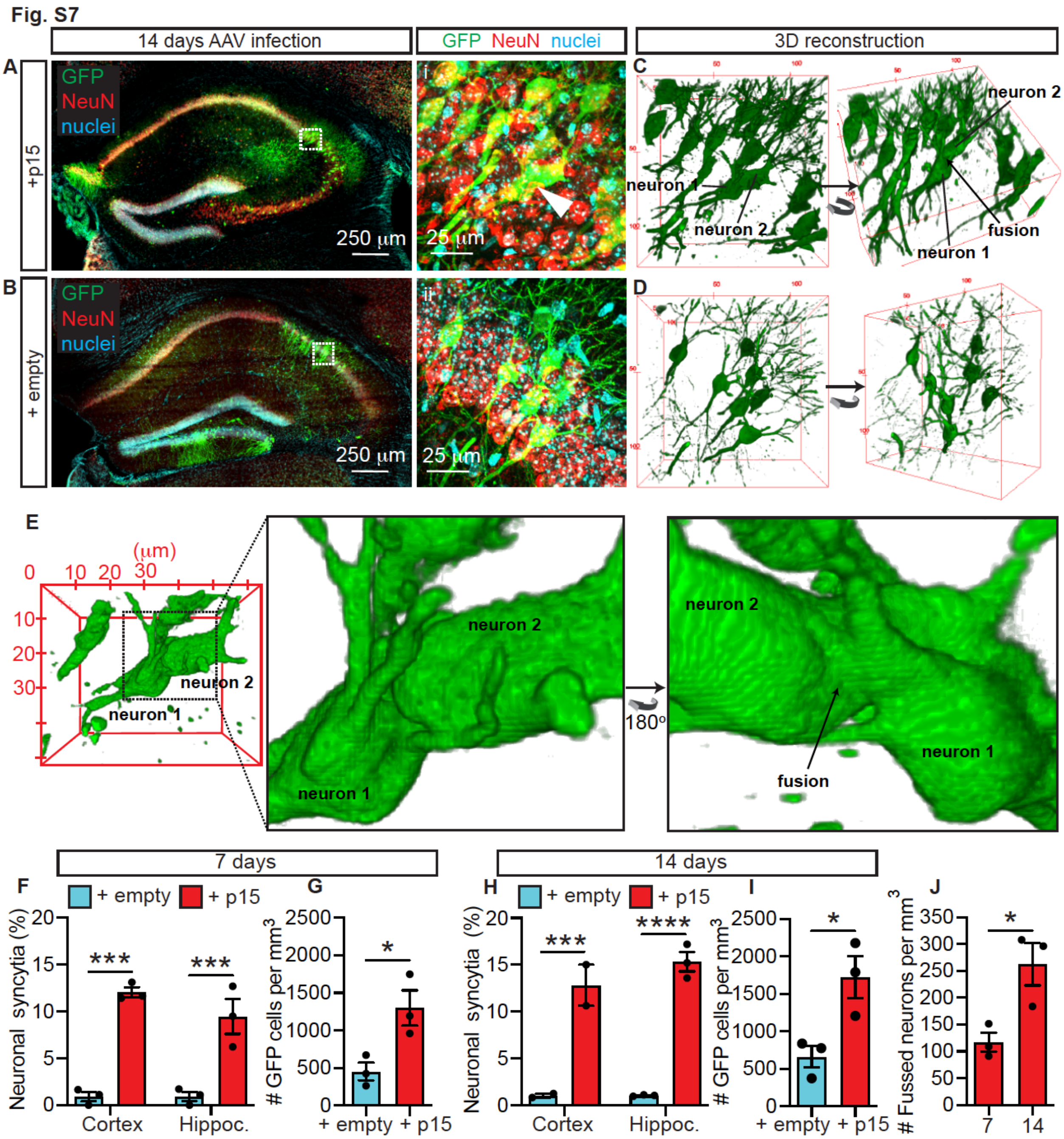

Fig. S7.

AAV transduction of p15 in adult murine brain mediates fusion of neurons in the cortex and the hippocampus.

(**A**, **B**), Representative images of the hippocampi of adult mice 14 days post-infection with AAV transducing under the *hSyn* promoter either p15 and GFP (a) or only GFP (empty vector) (b). Boxed areas are shown at higher magnifications in the right panels (i, ii), with P15 and GFP expressing neurons (arrowhead) fused at the level of their somas (panel i) versus non-fused GFP-neurons (panel ii). (**C**, **D**), Volumetric 3D reconstruction of fused neurons of panel i expressing both p15 and GFP (C), or non-fused GFP-neurons of panel ii expressing only GFP (D). (**E**),

Detail of the volumetric 3D reconstruction in (C). The boxed area is shown at higher magnification in the right panels. (**F**), Quantification of neuronal syncytia in the hippocampi and cortex of mice 7 days post-infection, measured as the percentage out of the total number of GFP-positive neurons. (**G**), Quantification of the number of GFP-positive neurons per volume in the hippocampi of mice 7 days post-infection. (**H**), Quantification of neuronal syncytia in the hippocampi and cortex of mice 14 days post-infection, measured out of the total number of GFP-positive neurons. (**I**), Quantification of the number of GFP-positive neurons per volume in the hippocampi of mice 14 days post-infection. (**J**), Quantification of the number of fused GFP-neurons per volume, 7 and 14 days post-infection with AAV p15 and GFP. Images in (A) and (B) show immunocytochemistry for nuclei (DAPI, blue), neuronal marker (NeuN, red), and GFP (green). 3D images in (C), (D) and (E) show two different orientations of the volumetric reconstructions. The two fused neurons in the panels (C) and (E) are indicated (neuron 1 and neuron 2), and the region where the neurons are fused (fusion) is highlighted with an arrow. Data in (F) to (J) are displayed as mean ± SEM. n = 3 infected animals per condition. Between 488 and 3069 GFP-neurons were quantified through the hippocampus and the cortex from AAV infected brains. The volume of tissue analysed ranged from 0.5 to 2 mm^3. Two-way ANOVA with Šidák correction for multiple comparisons were used in (F) and (H), and unpaired two-tailed Welch's *t* tests were used in (G), (I) and (J). *$p \leq 0.05$, ***$p < 0.001$, ****$p \leq 0.0001$.

Fig. S8

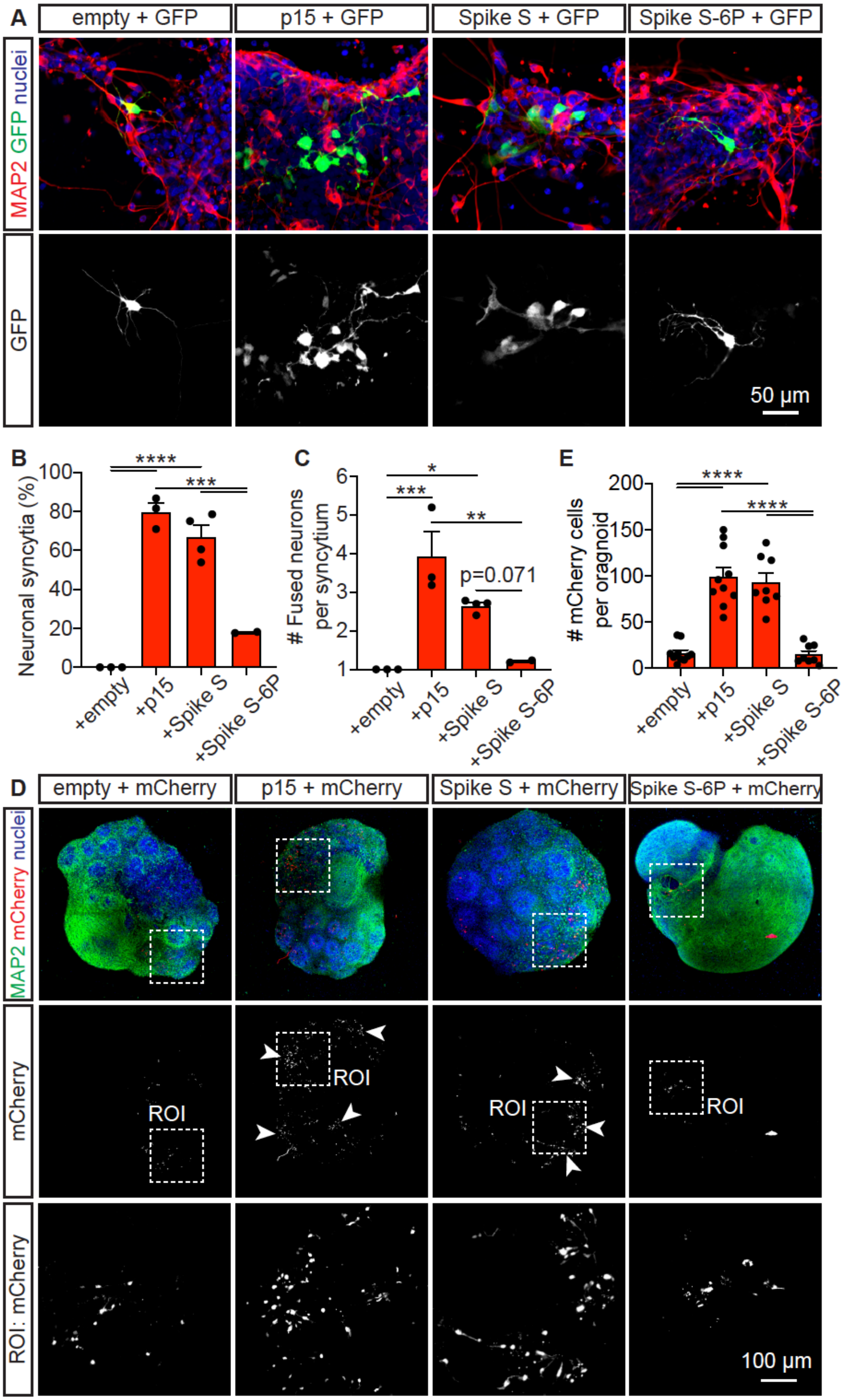

A
empty + GFP
p15 + GFP
Spike S + GFP
Spike S-6P + GFP
MAP2 GFP nuclei
GFP
50 µm
B
Neuronal syncytia (%)
****
***
+empty
+p15
+Spike S
+Spike S-6P
C
Fused neurons per syncytium
*
***
**
p=0.071
+empty
+p15
+Spike S
+Spike S-6P
E
mCherry cells per oragnoid
****
****
+empty
+p15
+Spike S
+Spike S-6P
D
empty + mCherry
p15 + mCherry
Spike S + mCherry
Spike S-6P + mCherry
MAP2 mCherry nuclei
mCherry
ROI
ROI: mCherry
100 µm

Fig. S8.

p15 and spike S induce fusion in human neurons and brain organoids.
(**A**), Representative images of 2D-cultured hESC-derived neurons illustrating fusion of cells into syncytia. Human neurons were co-transfected at 40-50 DIV with GFP and either p15, spike S, spike S-6P, or empty vector in controls, and then cultured for an additional 7 days. Immunocytochemistry for nuclei (blue), MAP2 (red), and GFP (green/white). (**B**), Quantification of neuronal syncytia as the percentage of interconnected neurons within a distance of ≤ 200 µm. (**C**), Quantification of the average number of interconnected neurons per syncytium containing more than one neuron. (**D**), Representative images of 3D hESC-derived brain organoids illustrating fusion of cells into syncytia. Brain organoids were co-transfected at 50 DIV with mCherry and either p15, spike S, spike S-6P, or empty vector in controls, and then cultured for 6 additional days. Immunocytochemistry for nuclei (blue), MAP2 (green) and mCherry (red/white). Regions of interest (ROIs) show higher magnification at positions indicated by broken lines. Arrowheads indicate clusters of fused neurons. (**E**), Quantification of the average number of mCherry-positive cells per organoid section 6 days after transfection. Data in (B), (C) and (E) are displayed as mean ± SEM, with averages of n > 30 neurons analyzed in independent experiments for (B) and (C), and n > 8 organoids analyzed in independent experiments for (E). One-way ANOVA followed by Tukey's *post hoc* test was used in (B-C). **p* <0.05, ***p* <0.01, ****p* <0.001, *****p* <0.0001.

Fig. S9

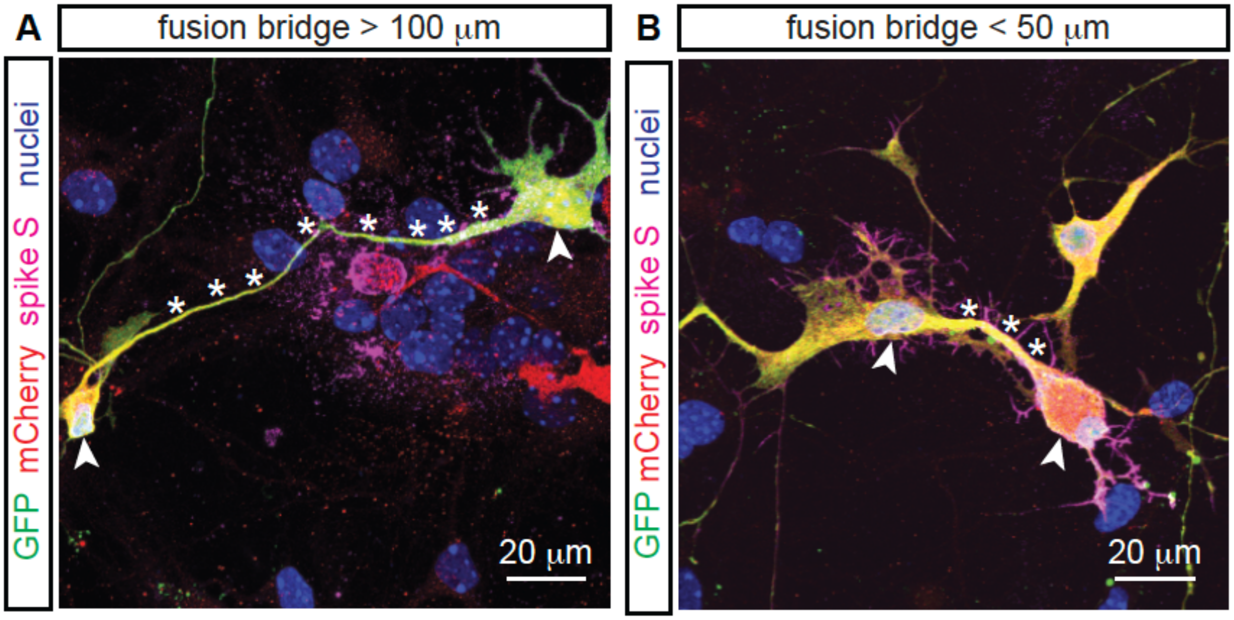

Fig. S9.

SARS-CoV-2 infection induces the fusion of neurons through long neuronal bridges.
(**A**), Representative image of SARS-CoV-2-infected and fused neurons (arrowheads) connected through a neuronal bridge (asterisks) that extends over 100 μm. (**B**), Representative image of SARS-CoV-2-infected and fused neurons (arrowheads) connected through a neuronal bridge (asterisks) that extends below 50 μm. Immunocytochemistry for nuclei (blue), GFP (green), mCherry (red) and spike S (magenta).

Fig. S10

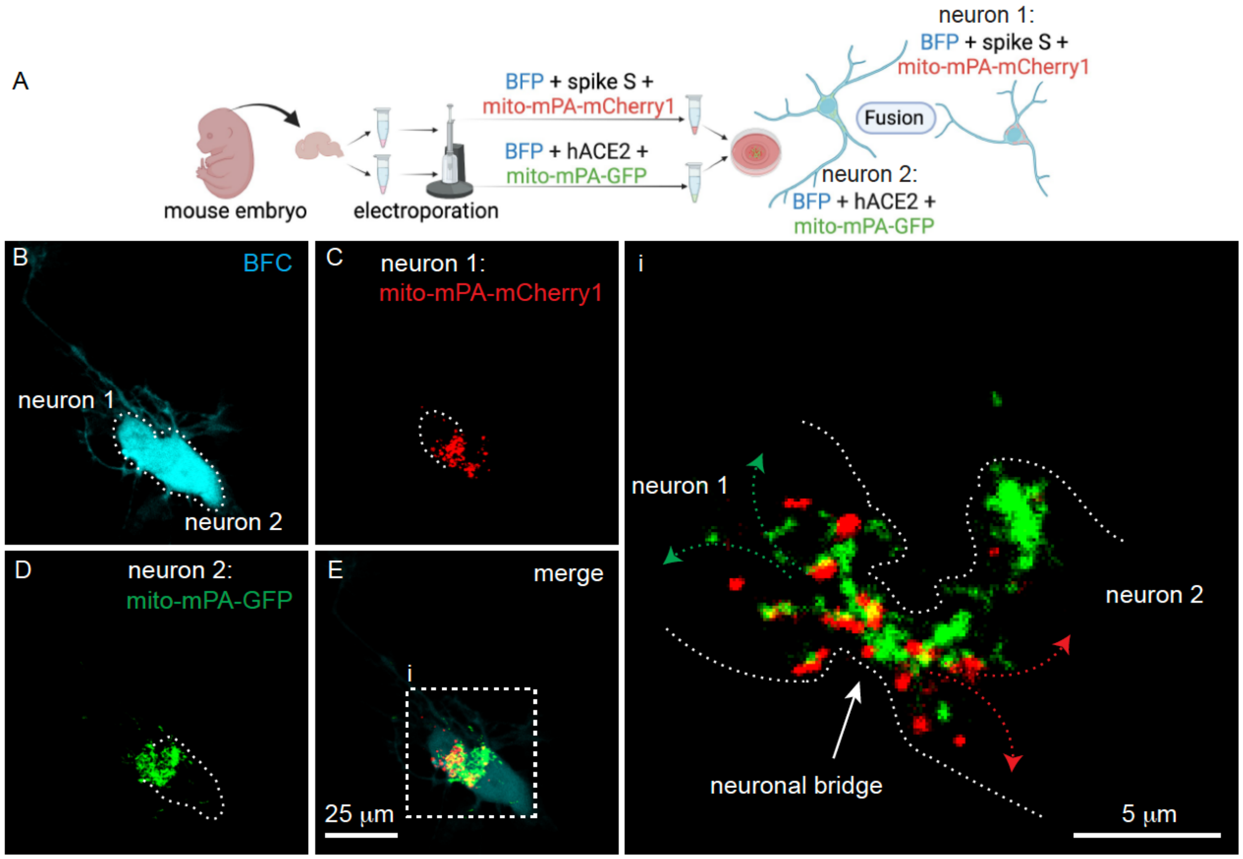

Fig. S10.

Bidirectional transfer of mitochondria between fused neurons.

(**A**), Scheme of the mitochondria exchange fusion assay. Hippocampi were dissected from the brains of mouse embryos, and two independent populations of hippocampal neurons were electroporated with either Spike S, BFP and mito-mPA-mCherry 1 or with hACE2, BFP, and mito-mPA-GFP. After electroporation, the neurons were mixed and cultured together. 5 days later the neuronal cultures were imaged. (**B-E**), Representative images of fused murine hippocampal neurons at 5 DIV expressing BFP (B), mito-mPA-mCherry (C), mito-mPA-GFP (D); merged images are in (E), with high magnification area in (i). The bidirectional transfer of mitochondria between neuron 1 and neuron 2 along the neuronal bridge is indicated with red and green arrows.

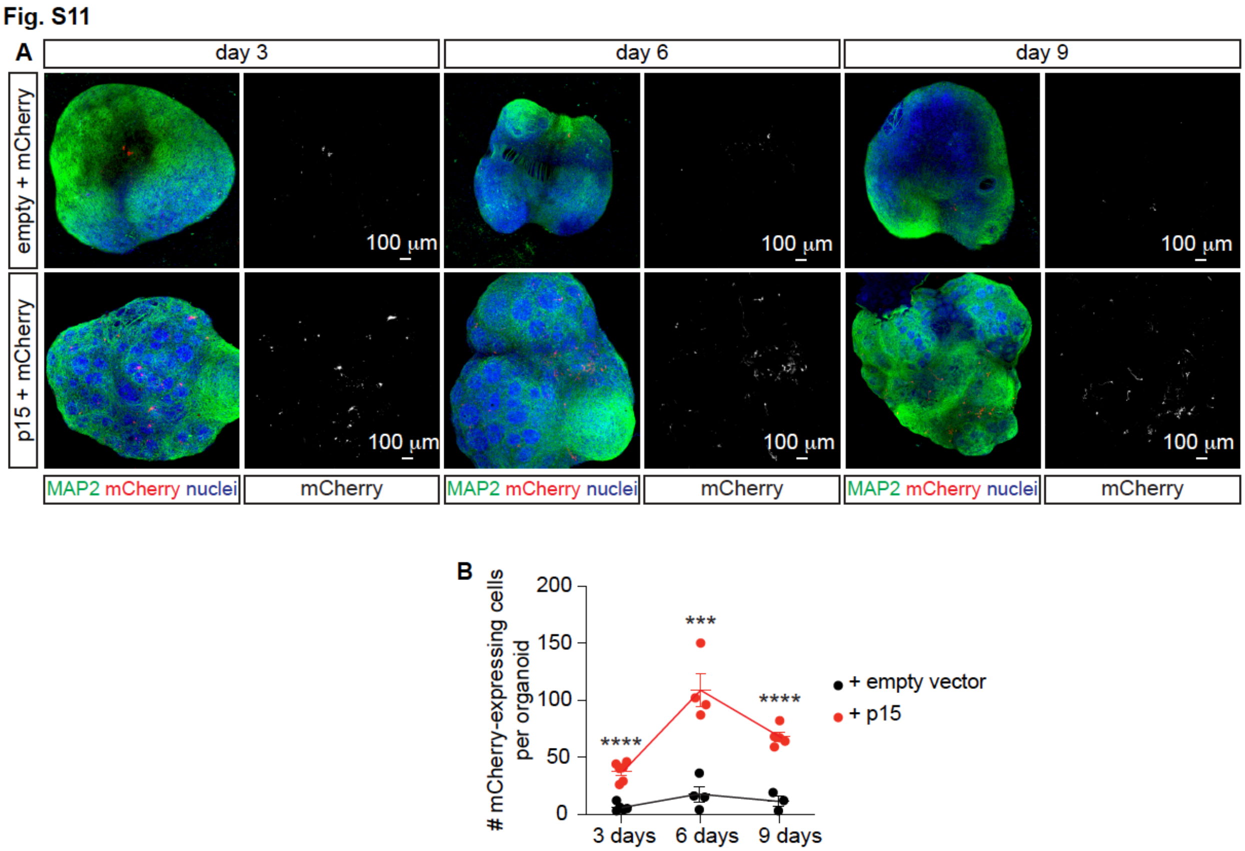

Fig. S11.

p15-induced neuronal fusion in human brain organoids is progressive with increased neuronal clusters.

(**A**), Representative images of 3D neuronal organoids illustrating fusion of cells into syncytia over time. Organoids were co-transfected at 43-50 DIV with mCherry and p15 (or empty vector in controls) and were then cultured for 3, 6 or 9 days. Immunocytochemistry for nuclei (blue), MAP2 (green) and mCherry (red/white). (**B**), Quantification of the average number of mCherry-positive cells per organoid 3, 6 and 9 days after transfection. Data in (B) are displayed as mean ± SEM. One-way ANOVA followed by Tukey *post hoc* test. ***p <0.001, ****p <0.0001.

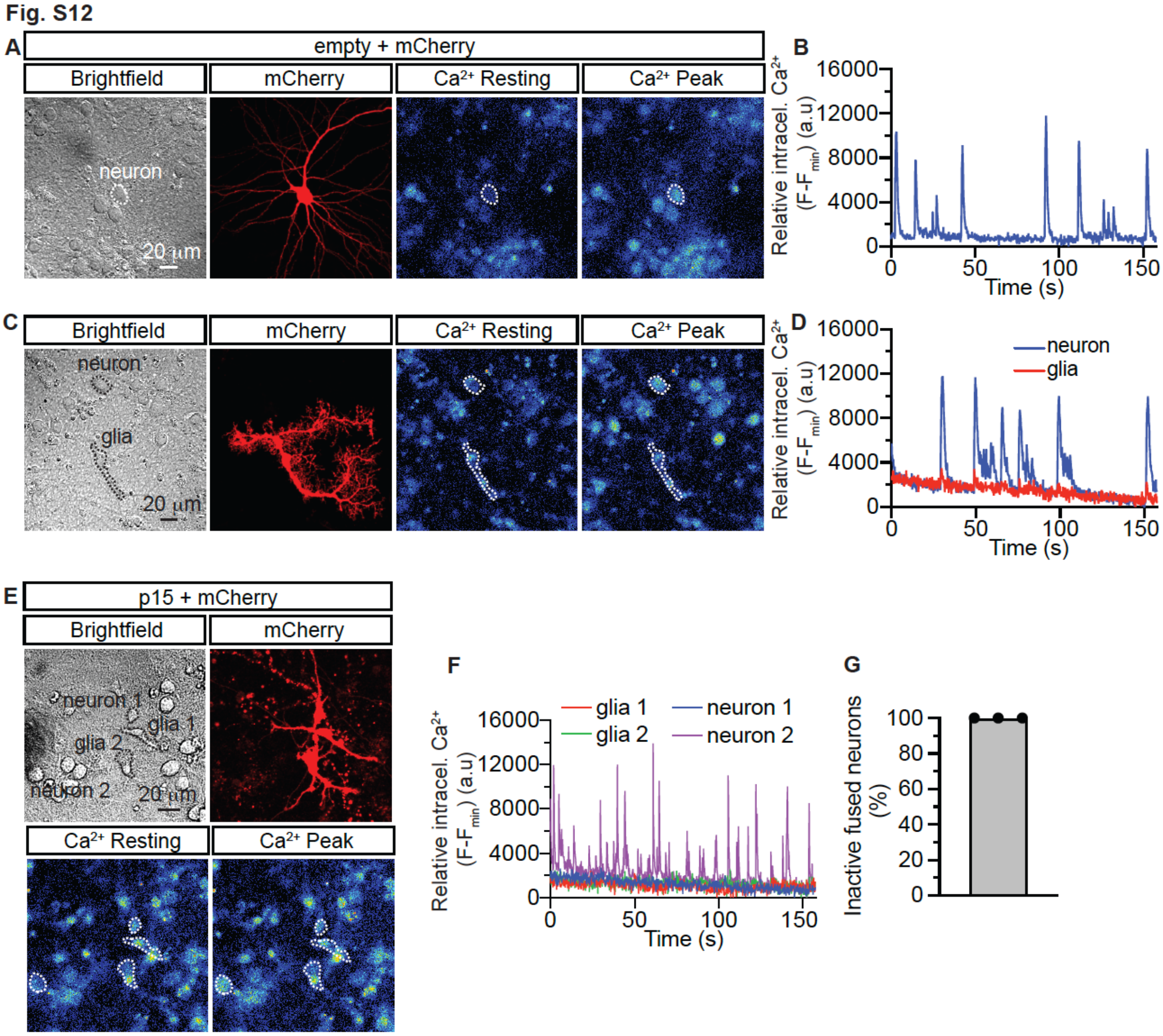

Fig. S12.

Neuronal-glia fusion completely abolishes neuronal activity.

(**A**), Representative images of a murine hippocampal neurons transfected at 12 DIV with mCherry, and imaged at 15 DIV after being incubated with CAL-520. The region of interest (ROI) through the images indicates the position of an mCherry-positive neuronal soma. The second and third panels show pseudocolored images of the intracellular Ca^{2+} levels in representative resting conditions and during an activity peak. (**B**), Plot of the relative intracellular Ca^{2+} intensity levels over time from the mCherry-positive neuron in panel (A). (**C**), Representative images of a murine glial cell transfected at 12 DIV with mCherry, and imaged at 15 DIV after being incubated with CAL-520. The elongated ROI through the images indicates the position of the mCherry-positive cell body of the glial cell. The rounded ROI indicates the position of the cell body of a non-transfected neuron. The second and third panels show pseudocolored images of the intracellular Ca^{2+} levels in representative resting conditions and during an activity peak. (**D**), Plot of the relative intracellular Ca^{2+} intensity levels over time from the mCherry-positive glial cell and mCherry-negative neuron of panel (C). Note the absence of

intracellular Ca^{2+} in the glial cell. (**E**), Representative images of one murine hippocampal neuron and two glial cells fused together. The hippocampal culture was co-transfected at 12 DIV with p15 and mCherry, and at 15 DIV after being incubated with CAL-520. The ROIs through the images indicate the position of the mCherry-positive neuron soma (neuron 1) and glial cell bodies (glia 1 and glia 2). The location of the soma of a non-transfected neuron is indicated with a fourth ROI (neuron 2). The panels below show pseudocolored images of the intracellular Ca^{2+} levels in representative resting conditions and during an activity peak. (**F**), Plot of the relative intracellular Ca^{2+} intensity levels over time from all the cells identified in panel (E). (**G**), Quantification of the percentage of observed inactive neurons fused with glial cells. Data in (G) are displayed as mean ± SEM. The data were obtained from 12 neurons imaged in 3 independent experiments.

Fig. S13

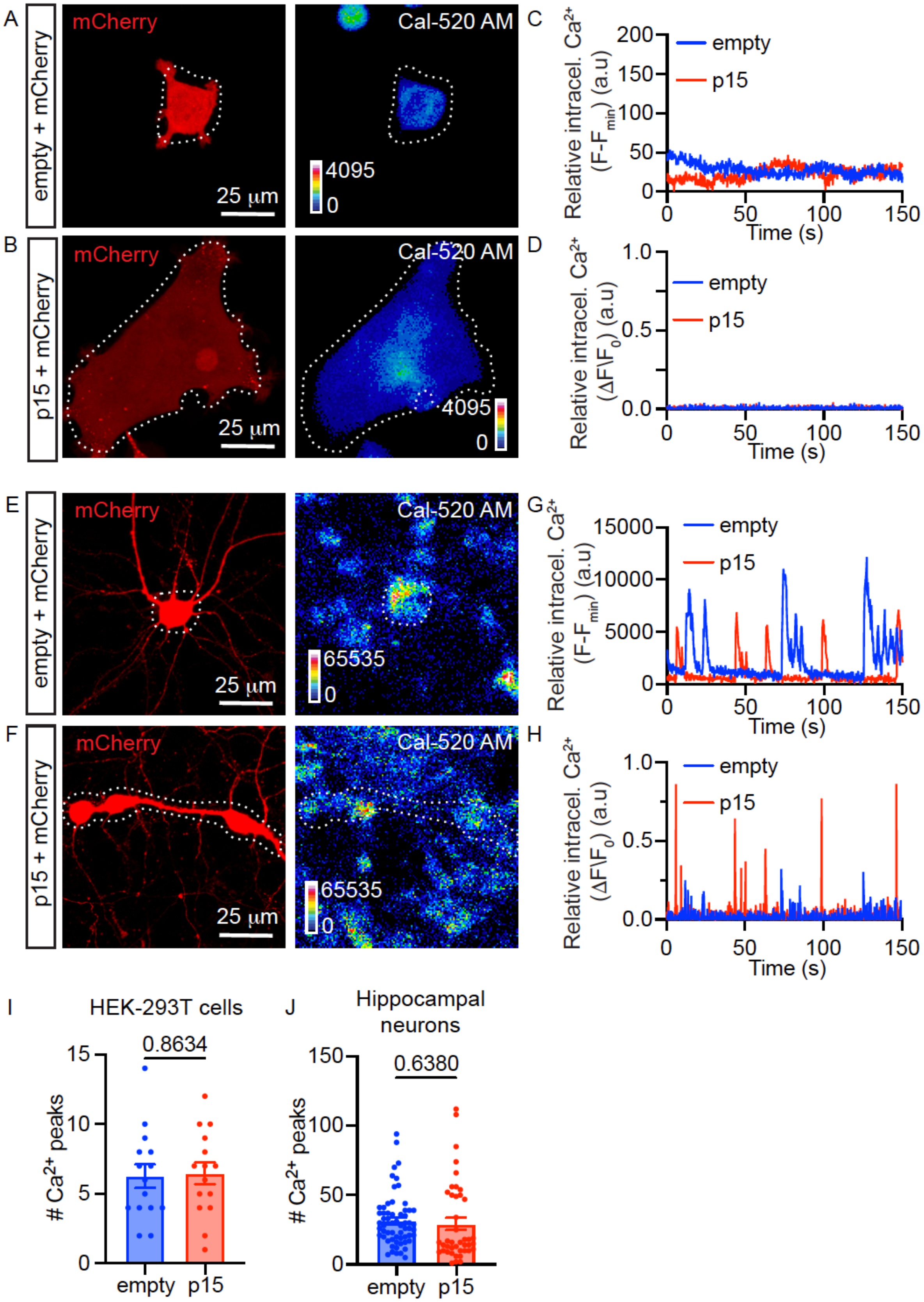

A
empty + mCherry
mCherry
25 µm
Cal-520 AM
4095
0
C
Relative intracel. Ca2+ (F-Fmin) (a.u)
empty
p15
Time (s)
B
p15 + mCherry
mCherry
25 µm
Cal-520 AM
4095
0
D
Relative intracel. Ca2+ (ΔF\F0) (a.u)
empty
p15
Time (s)
E
empty + mCherry
mCherry
25 µm
Cal-520 AM
65535
0
G
Relative intracel. Ca2+ (F-Fmin) (a.u)
empty
p15
Time (s)
F
p15 + mCherry
mCherry
25 µm
Cal-520 AM
65535
0
H
Relative intracel. Ca2+ (ΔF\F0) (a.u)
empty
p15
Time (s)
I
HEK-293T cells
0.8634
Ca2+ peaks
empty
p15
J
Hippocampal neurons
0.6380
Ca2+ peaks
empty
p15

Fig. S13.

p15-induced neuronal fusion does not modify the frequency of neuronal activity.
(**A**, **B**) Representative images of HEK-293T cells transfected either with an empty plasmid and mCherry (A), or with p15 and mCherry (B), which induces the formation of a large syncytium. The region of interest (ROI) through the images indicates the position of the analyzed cells. (**C**, **D**) Plots of the relative intracellular Ca^{2+} intensity levels over time from the mCherry-positive cells in the panels (A) and (B). (**E**, **F**) Representative images of murine hippocampal neuronsstransfected at 12 DIV with mCherry, and imaged at 15 DIV after being incubated with CAL-520. The region of interest (ROI) through the images indicates the position of an mCherry-positive neuronal soma. (**G**, **H**) Plots of the relative intracellular Ca^{2+} intensity levels over time from the mCherry-positive neurons in the panels (E) and (F). (**I, J**) Quantification of the number of intracellular Ca^{2+} peaks in HEK-293T cells (I) and in hippocampal neurons (J) fused by p15. Data in (I) and (J) are displayed as mean ± SEM. The data in (I) was obtained from 15 cells imaged in 3 independent experiments, whereas the data in (J) was obtained from 42-61 neurons in over 3 independent experiments. Unpaired two-tailed Welch's *t* tests was used. P-value is indicated.

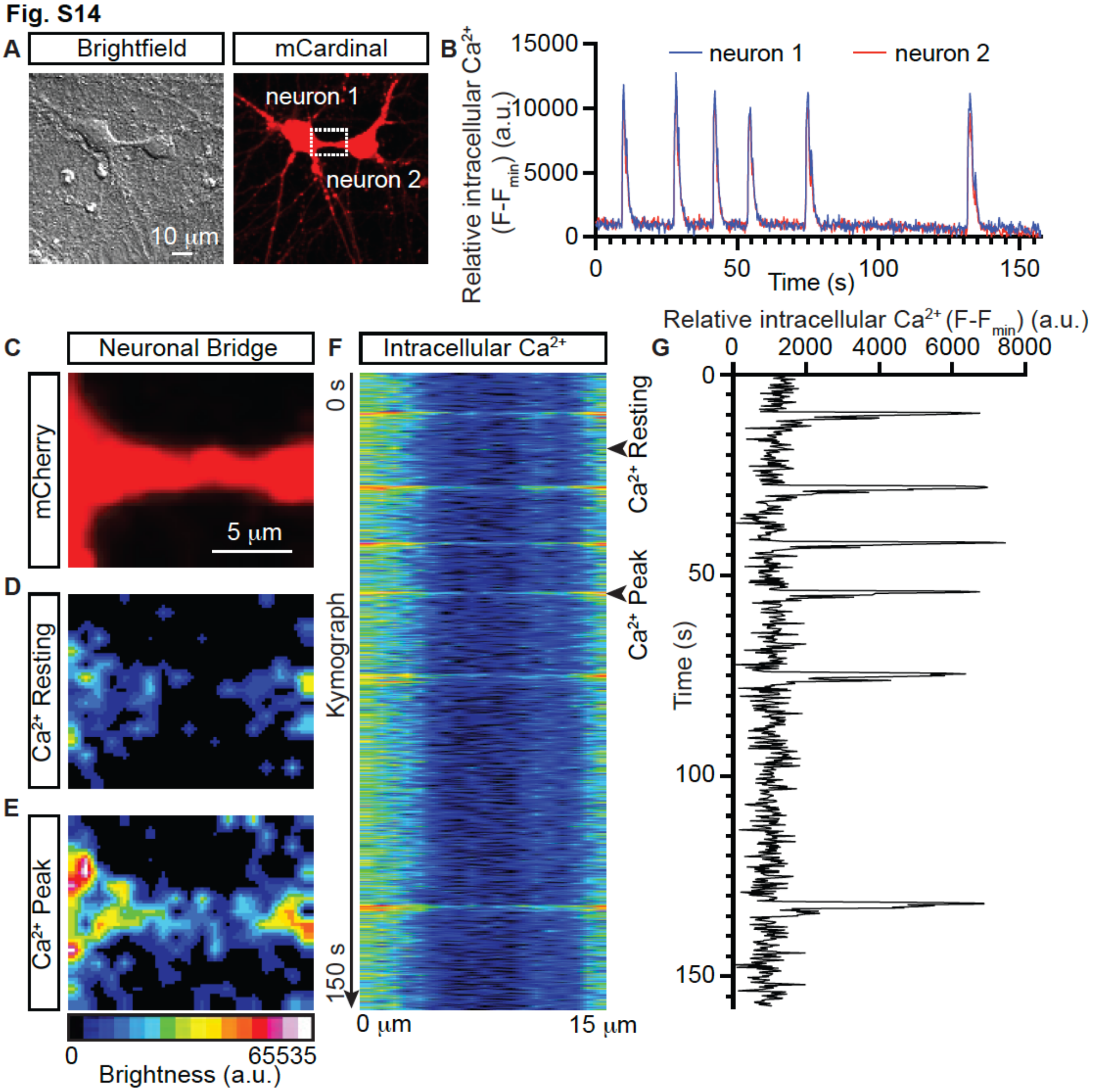

Fig. S14.

Neuronal activity is preserved within the cytoplasmic bridges of fused neurons.
(**A**), Representative images of two fused murine hippocampal neurons that were co-transfected at 12 DIV with mCherry, and imaged at 15 DIV after being incubated with CAL-520. (**B**), Plot of the relative intracellular Ca^{2+} intensity levels over time of the mCherry-positive fused neuron 1 and neuron 2 from panel (A). (**C**), Detail of the boxed region of interest (ROI) in (A), corresponding to the neuronal bridge that connects the fused neuron 1 and neuron 2. (**D**), Pseudocolored image of the intracellular Ca^{2+} levels observed within the neuronal bridge in representative resting conditions. (**E**), Pseudocolored image of the intracellular Ca^{2+} levels observed within the neuronal bridge in representative activity peak conditions. (**F**), Kymograph of the pseudocolored images of the intracellular Ca^{2+} levels obtained within the boxed ROI in (A) (neuronal bridge) during 157.4 s. (**G**), Plot of the relative intracellular Ca^{2+} intensity levels along time of the boxed ROI in (A) (neuronal bridge).

Movie S1.

Photoconverted red Kaede diffuses between p15-fused neurons. Movie at 20 frames per second (fps), with frames being acquired every 785 ms. 5 frames were acquired before photoconversion and 50 frames after photoconversion. Photoconversion was performed by applying a UV pulse on a 5 μm x 5 μm region of interest (ROI, white square). Scale bar represents 20 μm.

Movie S2.

Volumetric 3D reconstruction of fused neurons shown in Fig. S6 E. 720° rotation on the vertical axis. The first 360° rotation represent the volumetric reconstruction of the GFP image. The following 360° rotation represent the volumetric reconstruction of both the GFP image and the nuclei image. Note that the GFP intensity has been reduced to facilitate the appreciation of the two nuclei inside the two fused neurons.

Movie S3.

Mitochondria diffuse between p15-fused neurons. Movie at 5 fps, with frames being acquired every 5 min. Images were recorded 10 min after mito-mPA-GFP photoactivation.

Movie S4.

Neuronal fusion is progressive and generates a multicellular neuronal syncytium. Movie at 10 fps, with frames being acquired every 30 min. Images were recorded 24 h after transfecting hippocampal neurons with p15 and GFP. Each frame represents the maximum intensity projection, with GFP intensity adjusted to facilitate the visualization of newly appearing GFP-positive neurons. Scale bar represents 50 μm.
Scale bar for the color-coded intracellular Ca^{2+} levels:

0 65535

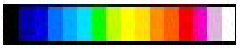

Movie S5.

Spontaneous intracellular Ca^{2+} levels in p15-fused neuron. Pseudocolored video indicating intracellular Ca^{2+} levels. Movie at 5 fps, with frames being acquired every 0.197 s. The movie corresponds to the neuron in Fig. 4A. The neuron was transfected with p15 and mCherry and imaged 3 days later at 15 DIV after being incubated with CAL-520.
Scale bar for the color-coded intracellular Ca^{2+} levels:

0 65535

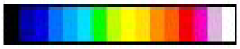

Movie S6.

Spontaneous intracellular Ca^{2+} levels in non-fused control neuron. Pseudocolored video indicating intracellular Ca^{2+} levels. Movie at 5 fps, with frames being acquired every 0.197 s.

The movie corresponds to the neuron in Fig. 4B. The neuron was transfected with mCherry and imaged 3 days later at 15 DIV after being incubated with CAL-520.
Scale bar for the color-coded intracellular Ca^{2+} levels:

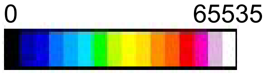

Movie S7.

Spontaneous intracellular Ca^{2+} levels within the neuronal bridge connecting two p15-fused neurons. Pseudocolored video indicating intracellular Ca^{2+} levels. Movie at 5 fps, with frames being acquired every 0.197 s. The movie corresponds to the neuron in Fig. S10a. The neuron was transfected with p15 and mCherry and imaged 3 days later at 15 DIV after being incubated with CAL-520.
Scale bar for the color-coded intracellular Ca^{2+} levels:

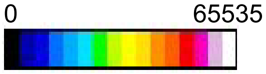

Supplement: Supplementary file 1 — Figs. S1 to S14 Legends for movies S1 to S7 [file sciadv.adg2248_sm.pdf]
